# Premature termination codons in modern human genomes

**DOI:** 10.1038/srep22468

**Published:** 2016-03-02

**Authors:** Kohei Fujikura

**Affiliations:** 1 Kobe University School of Medicine, 7-5-1, Kusunoki-cho, Chuo-ku, Kobe 650-0017, Japan

## Abstract

The considerable range of genetic variation in human populations may partly reflect distinctive processes of adaptation to variable environmental conditions. However, the adaptive genomic signatures remain to be completely elucidated. This research explores candidate loci under selection at the population level by characterizing recently arisen premature termination codons (PTCs), some of which indicate a human knockout. From a total of 7595 participants from two population exome projects, 246 PTCs were found where natural selection has resulted in new alleles with a high frequency (from 1% to 96%) of derived alleles and various levels of population differentiation (*F*_*ST*_ = 0.00139–0.626). The PTC genes formed protein and regulatory networks limited to 15 biological processes or gene families, of which seven categories were previously unreported. PTC mutations have a strong tendency to be introduced into members of the same gene family, even during modern human evolution, although the exact nature of the selection is not fully known. The findings here suggest the ongoing evolutionary plasticity of modern humans at the genetic level and also partly provide insights into common human knockouts.

Understanding the spectrum of allelic variation in human genes and revealing the demographic and evolutionary forces that shape this variation within and among populations are major aims of human genetics research. The intuitive view that natural selection has ceased to affect modern humans has been frequently encountered in the past^1^,^2^. Yet, recent genetic studies demonstrate that some genes exhibit signs of recent, strong selection in favor of new alleles^3^,^4^,^5^,^6^,^7^,^8^,^9^. These include malaria resistance^3^,^4^, lactase persistence^5^, bitter taste change^6^, and reduced olfactory receptors^7^. These examples of recent selection in humans were discovered in studies of candidate genes with a prior hypothesis of selection^3^,^4^,^5^,^6^,^7^. More recently, whole-genome approaches have been used to identify candidate genomic loci that may be targets of positive selection during human evolution^8^,^9^. However, much is still unknown about the types of genes or biological processes commonly involved in the adaptation of modern humans.

There are several major theories of mutation-driven evolution^10^,^11^,^12^. The theory that gene duplication is a primary driving force in shaping molecular evolution was proposed in the 1970s^10^ and is now widely accepted. The paradoxical idea that gene loss events can also greatly facilitate adaptive evolution was also proposed^11^. Common sense may lead us to perceive gene loss as a deleterious change and to associate adaptation with benign mutations. However, as James Neel proposed, thrifty genotypes that were advantageous for hunter-gatherer populations may be detrimental in modern times (Thrifty gene hypothesis)^12^.

The true effects of non-synonymous mutations are generally difficult to evaluate without functional analysis. One clear signature of gene evolution is the introduction of premature termination codons (PTCs)^13^. PTC can result in truncated gene products and some may be regarded as equivalent to human knockouts, as is the case with model organisms. In addition, shorter proteins are usually eliminated by a post-transcriptional process termed nonsense-mediated mRNA decay (NMD)^14^,^15^. If the PTC allele is harmful to its carrier, it will likely be eliminated by the forces of negative selection, whereas positive selection will act to increase its frequency should the allele be advantageous^16^,^17^. When the mutation is neutral, it can either increase or decrease its frequency in a population through the random effects of genetic drift^16^.

The pioneering work by Wang *et al*. identified 80 human-specific non-processed pseudogenes and provided population-genetic (popgen) data on the adaptive loss of *CASP12*^18^. Later on, Yngvadottir *et al*. genotyped 805 PTCs and validated 169 of them^19^. They found that more than 10 of them (including *CASP12*) were prone to adaptive selection as revealed by popgen parameters or tests (extreme *F*_*ST*_, DAF, etc.)^19^. MacArthur *et al*. then applied filters to 2951 putative loss-of-function variants obtained from 185 human genomes and validated 565 out of 1111 PTCs^20^. They discovered that 20 out of 565 PTCs may be subject to selection, as revealed by popgen tests^20^. In this current research, a further large-scale attempt was performed to investigate the recently arisen PTCs using two population exome projects.

## Results

### Identification of premature termination codons (PTCs)

**Figure 1:**
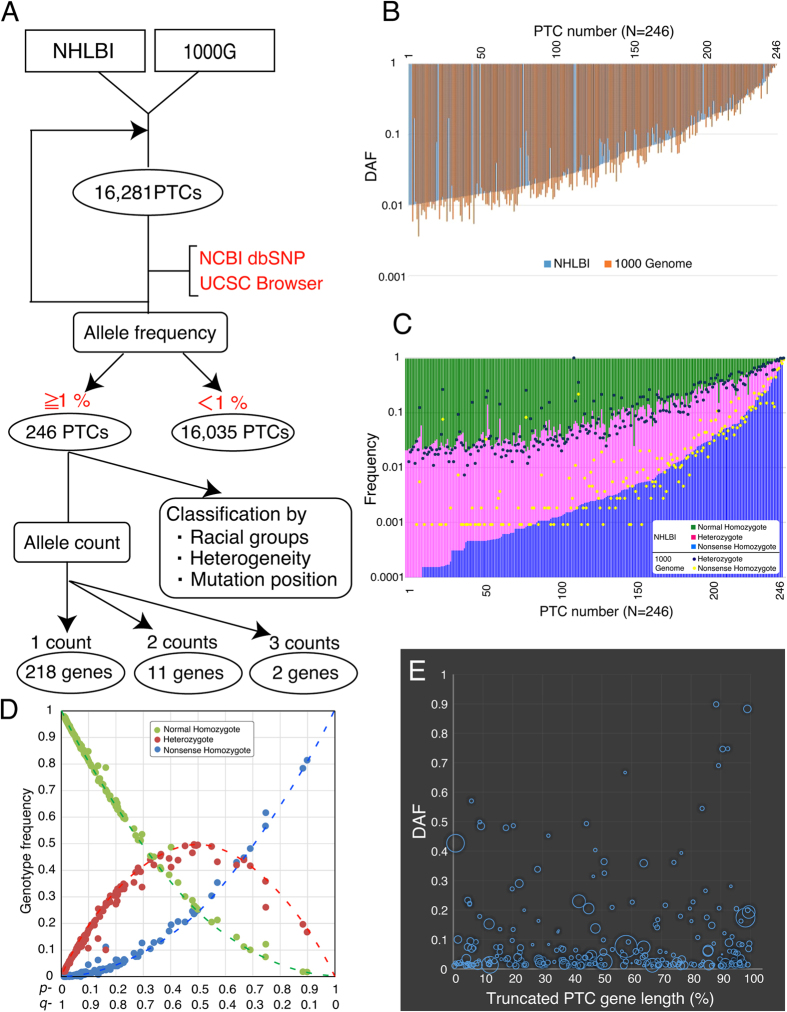
Identification and annotation of PTC mutations with a DAF >1%. (**A**) Flow chart used to identify and classify the PTC mutations that interrupt open reading frames, showing 16,281 mutations within the NHLBI and 1000G projects. These mutations include small proportions of variants with error, and thus several platforms (NCBI dbSNP and UCSC Browser) were used to access the validity of variants and were also employed for precise annotation (gene name, accession number, ancestral alleles, dbSNP ID). PTC mutations were then classified according to allele frequency. (**B**) Allele frequency distribution of all PTC mutations found in the NHLBI database (blue) and 1000G project (orange) plotted on a logarithmic scale. PTC mutations are sorted along the x-axis according to frequency of nonsense homozygotes. (**C**) Genotype frequencies of normal homozygotes (NHLBI: green), heterozygotes (NHLBI: purple, 1000G: dark blue dot), and nonsense homozygotes (NHLBI: blue, 1000G: yellow dot) for each PTC plotted on a logarithmic scale. (**D**) Three observed genotypes (normal homozygosity (green), heterozyosity (red), nonsense homozygosity (blue)) were compared to those expected under Hardy-Weinberg equilibrium (dotted line). The horizontal axis shows two allele frequencies *p* and *q*, and the vertical axis shows the genotype frequencies. (**E**) Distributions of truncated gene length (%) (horizontal axis), DAF (vertical axis), and gene size (circular size) of 246 PTC mutations. Regression equation with a 95% confidence dashed line is shown in yellow.

Two large sets of population exome sequences, from the National Heart, Lung, and Blood Institute (NHLBI)^21^ and the 1000 Genomes (1000G) Project^22^, were utilized to identify recently arisen PTCs in the human genome (Fig. 1A). The systematic search yielded 16,281 segregating PTCs from a total of 7,595 individuals selected from 16 ethnic populations (Fig. 1A). Information on alleles containing PTC mutations was retrieved from the NCBI dbSNP and UCSC genome browser. A derived allele frequency (DAF) of 1% was targeted as a reference point. Based on this criterion, 246 PTCs in 231 genes were selected for further analysis (Figs 1A and S1, Table S1). These PTC mutations may reflect one aspect of recent genomic alternations in modern humans. The majority of genes were non-repetitive (218/231; 94.4%), two genes (PKD1L2 and C17orf57) had three counts (2/231; 0.9%) and the remaining 11 genes (*ABCA10, C18orf56, CCHCR1, FAM187B, FAM187B, OR7G3, PKD1L2, RFPL1, SLC22A24, SPERT, ZNF80*) had 2 counts each (11/231; 4.8%) (Figure S1). This result showed the strong mutational biases in modern human genomes. The DAF distribution of PTCs from NHLBI was indistinguishable from that of the 1000G (Wilcoxon signed-rank test; *p* = 0.853) (Fig. 1B), and there were no significant differences in the heterozygous/homozygous state of PTCs between the two population exomes (Wilcoxon signed-rank test; Normal homozygote *p* = 0.436, Normal/PTC heterozygote *p* = 0.259, PTC homozygote *p* = 0.527) (Fig. 1C). The distribution of PTC homozygotes (*f* (aa) = *q*^2^) was in Hardy-Weinberg equilibrium (Fig. 1D) (see also P-P plots in Figure S2). These results suggest two conclusions: (1) the possibility PTC homozygotes are not associated with decreased survival rates, and (2) that allele frequency is not changing rapidly from one generation to the next.

To examine the relationships between DAF, truncation position, and full-length open reading frame (ORF) of the selected PTCs and their effect on the gene products, the truncations were calculated as a percentage of the full-length ORF (Figs 1E and S3). The truncations were evenly distributed throughout the length of the ORF (Median: 48.9%; Deviation from unity: 1.12 ± 2.92%) and approximately 80% of PTCs led to the deletion of more than 20% of the amino acid sequence (Figure S3). A clear relationship between frequency and position of PTCs and ORF length was not statistically observed (Fig. 1E). These results suggest that most PTCs seriously alter gene length and this distribution cannot be explained by the meaningless shortening of the tail end of genes.

To assess the pathogenicity of 246 PTC mutations, two commonly used methods, SIFT^23^,^24^ and MutationTaster^25^ were applied to these variants. One-hundred percent (224/224) of variants were predicted as deleterious by SIFT, while 22 variants were not recognized (Table S2). MutationTaster was used to compensate the data and four variants, which were not recognized by SIFT, were newly predicted as disease-causing variants (Table S2). In theory, at least 118 variants undergo regulation of NMD, while the others are difficult to evaluate but may also cause NMD (Table S2).

### Functional classification of the PTC genes

**Figure 2:**
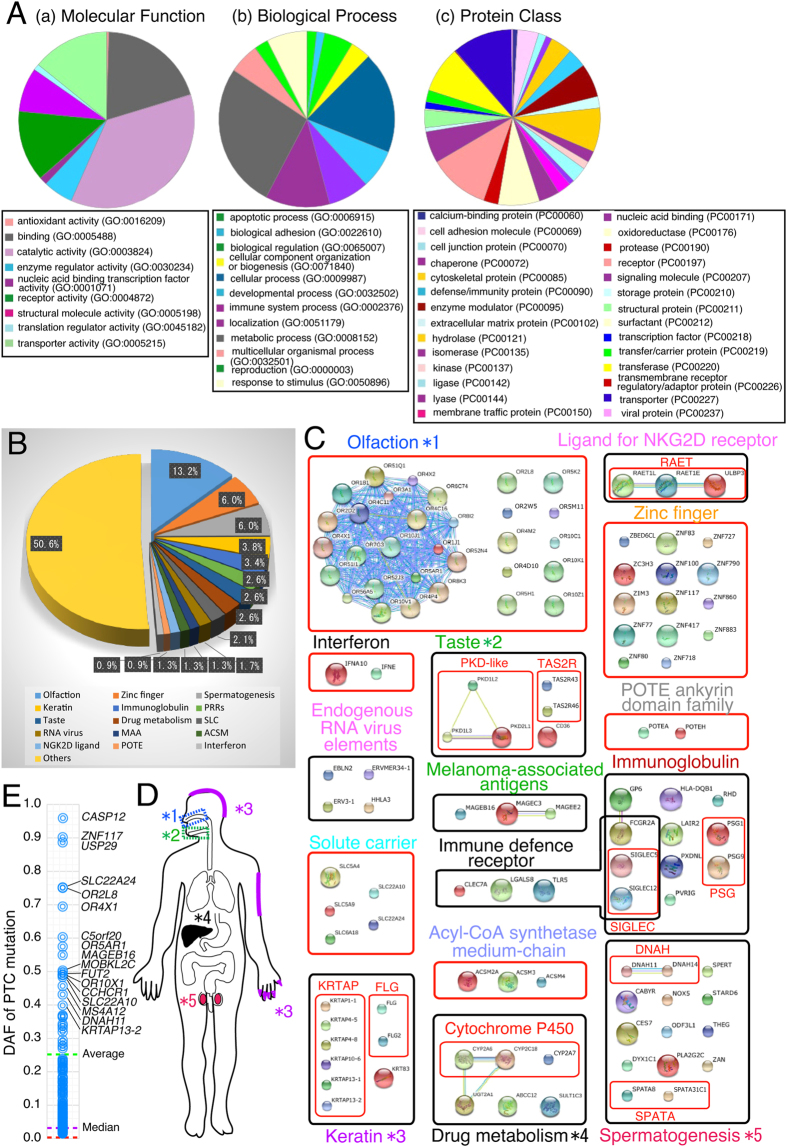
Functional bias of 235 PTC genes with a total DAF >1%. (**A**) Gene ontology (GO) distribution of gene functional annotations. GOs were derived from PANTHER version 9.0. Three different modes (Molecular Function, Biological Process and Protein Class) were used for the analysis. (**B**) Functional classification of 235 PTC genes. This chart was constructed using STRING. (**C**) The protein-protein interaction networks of the PTC genes were described using GO and STRING. Large circles indicate the structures of proteins and small circles represent structurally unidentifiable proteins. Black and red boxes indicate functional and structural classification, respectively. Color-coded lines represent various interactions: blue, binding; pink, post-translational modification; olive green, expression; red, inhibition; green, induction; and black, reaction. (**D**) Five systemically-classified groups in Fig. 2B are labeled with the colors of corresponding body parts. (**E**) Allele frequency distribution of 246 PTC mutations, showing the list of genes with frequently occurring PTCs. Green and purple lines indicate average and median frequency of mutations, respectively. The red line shows the average PTC frequency of all genes of the human genome calculated based on standards (~0.16%).

The functional links were then clarified among 235 genes that contain PTCs with a total DAF >1% (*In this section “235” genes were selected for functional classification because total PTC frequency per gene is important for the gene-by-gene analysis; see also Figure S1). In the first step, the Gene Ontology (GO) repository^26^ of the 235 genes was surveyed on the basis of molecular function (Fig. 2A, left panel), biological process (middle panel), and protein class (right panel). The functional interactome of the proteins was explored utilizing STRING software^27^ and integrated with GO classifications. A total of 15 biological processes were clearly established to accommodate the PTC genes emerging during human evolution (Fig. 2B,C). The largest proportion of genes were found to be functional for olfaction (13.2%), followed by zinc fingers (6.0%), spermatogenesis (6.0%), keratins (3.8%), taste (2.6%), ligands for NGK2D (1.3%), and others (Fig. 2B). Figure 2B shows 50.6% of genes did not form clusters, although some genes were functionally and/or structurally analyzed.

A total of 31 genes for olfaction were identified as recently mutated genes with DAF >1% (Fig. 2C,D). A reduction in the number of keratin-related genes, such as six KRTAPs, two FLGs, and *KRT83* (Fig. 2C,D) was also observed. These results are partly consistent with previous reports^28^, although there are differences in the variety and number of detected genes. A similar loss of genes for taste was found in three PKD-like proteins responsible for sour taste (members of a transient receptor potential (TRP) channel), two TAS2R proteins responsible for bitter taste, and one CD36 protein responsible for fat taste (Fig. 2C,D). The taste receptor has recently been shown to be one of the most pronounced cases of functional population diversity in the human genome^29^. Loss of the genes in the seven other categories were previously unreported (three NGK2D ligands, five solute carriers, three melanoma-associated antigens, three medium-chain Acyl-CoA synthetases, two POTE ankyrin domains, two interferons, and six immune defense receptors; Fig. 2C).

A DAF distribution of the 246 PTCs with the highest DAF (96.0%) for the PTC mutation in *CASP12* is shown in Fig. 2E. Other PTCs with relatively high DAFs were *ZNF117* (89.9%), *USP29* (88.3%), two solute carriers (*SLC22A24*, 74.7%; *SLC22A10*, 47.9%), four olfactory receptors (*OR2L8*, 74.8%; *OR4X1*, 69.1%; *OR5AR1*, 57.0%; *OR10X1* , 48.6%), and melanoma-associated antigens (*MAGEB16*, 54.5%). These frequencies were much higher than the overall ones in the human genome (approximately 0.16%; calculated from PTCs caused by single nucleotide substitution) (Fig. 2E, see also Table S1), but these PTC mutations still remain in a fluctuating state across the human genome.

### Inter-ethnic differences in PTC mutations

**Figure 3:**
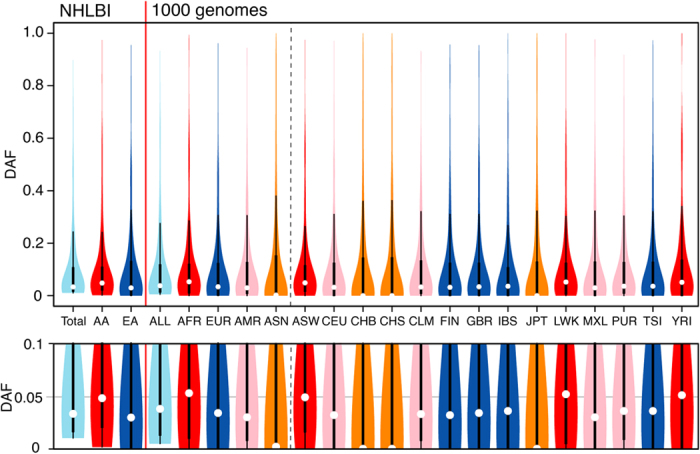
Violin plot analysis of 246 PTCs among 16 ethnic groups. Distribution of allele frequency of 246 PTC mutations in 16 samples (black line, first and third quartiles; white circle, median). The lower graph is an enlarged view of the upper panel. The NHLBI datasets include two ethnic origins (AA, African Americans; EA, European Americans) and 1000G datasets included 14 ethnic origins (ASW, American’s of African Ancestry in SW; CEU, Utah Residents (CEPH) with Northern and Western European ancestry ; CHB, Han Chinese in Beijing; CHS, Southern Han Chinese; CLM, Colombian from Medellin; FIN, Finnish in Finland; GBR, British in England; IBS, Iberian population in Spain; JPT, Japanese in Tokyo; LWK, Luhya in Webuye; MXL, Mexican ancestry from Los Angeles; PUR, Puerto Rico from Puerto Rica; TSI, Toscani in Italia; YRI, Yoruba in Ibadan).

Geographically separated populations are subject to distinctive selective environments^30^. Regional distribution of PTC mutations was next investigated by analyzing their frequencies and distributions. Principal component analysis (PCA) and hierarchical clustering analysis (HCA) of 246 PTCs among 16 ethnic populations clearly showed four rough subdivisions (AFR [AA, ASW, LWK and YRI], EUR [EA, CEU, FIN, GBR, IBS and TSI], ESN [CHB, CHS and JPT] and HIS [CLM, MXL and PUR]) of human PTC diversity (Figure S4). This ethnic difference is nearly consistent with some recent studies in overall site spectrum frequency (SFS), including synonymous and non-synonymous mutations^21^,^22^,^31^,^32^, while SFS limited to PTC mutations was not well described in previous studies. Violin plots of 246 PTCs across 16 populations showed that the median DAF for the variants in Africans (AFR: 0.053, AA: 0.048) was higher compared within Europeans (EUR: 0.034, EA: 0.030) and Hispanics (AMR: 0.030) (Fig. 3). East Asians had a unique allele frequency with a median DAF of almost zero (0.002) (Fig. 3). This result suggests that non-African populations have more PTC variants with a higher DAF per individual compared with Africans, and Asians have the largest number of high-frequency PTC alleles.

To address the origin of nonsense codons, the acquisition of 246 PTCs in modern humans was compared to the genomes of the archaic humans Neanderthals^33^ and Denisovans^34^, likely sister groups to modern humans. Virtual genotyping illustrated that 21.9% (54/246) of PTC alleles were detected in either Denisovan or Neanderthal genomes, although further population-scale research is necessary. This result suggests that the identified decrease in the functional gene repertoire is a relatively recent genomic process.

**Figure 4:**
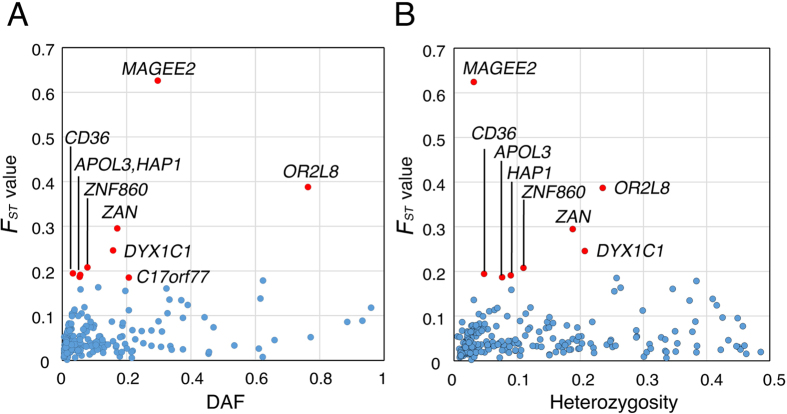
Population divergence as measured by *F*_*ST*_ values of 246 PTCs. The *F*_*ST*_ values of 246 PTCs with DAF >1% among 14 ethnic populations are plotted against their DAF (**A**) and heterozygosity (**B**) in blue. The outliers (standard residuals above +2) were detected by regression analysis and labeled with red circles.

The degree of population differentiation among 14 ethnic populations was then calculated as an *F*_*ST*_ value^35^. PTC alleles with DAF >1% had low to high *F*_*ST*_ values within the 0.00139–0.626 bin (Table S1). The *F*_*ST*_ values of PTCs were plotted against their DAF and no significant correlation was observed between the two (Fig. 4A). However, the outlying *F*_*ST*_ values were mainly detected in PTCs with moderate and high DAFs (Fig. 4A). Therefore, the moderate and high *F*_*ST*_ values observed for the PTCs here are likely to be a consequence of selection acting differently across different sub-populations. Use of other distance measures (*G’*_*ST*_^36^ and *Jost’s D*^37^) gave similar results (Figure S5 and Table S1).

*F*_*ST*_ was also compared to heterozygosity (Fig. 4B). No overall correlation was found between the two, but it should be noted that several PTCs displaying relatively high *F*_*ST*_ values showed outlier behavior (i.e., lying above the 95^th^ percentile) (Fig. 4B), which could indicate a recent population-restricted selective sweep.

### Geographical distribution of the 15 categories of PTC genes

**Figure 5:**
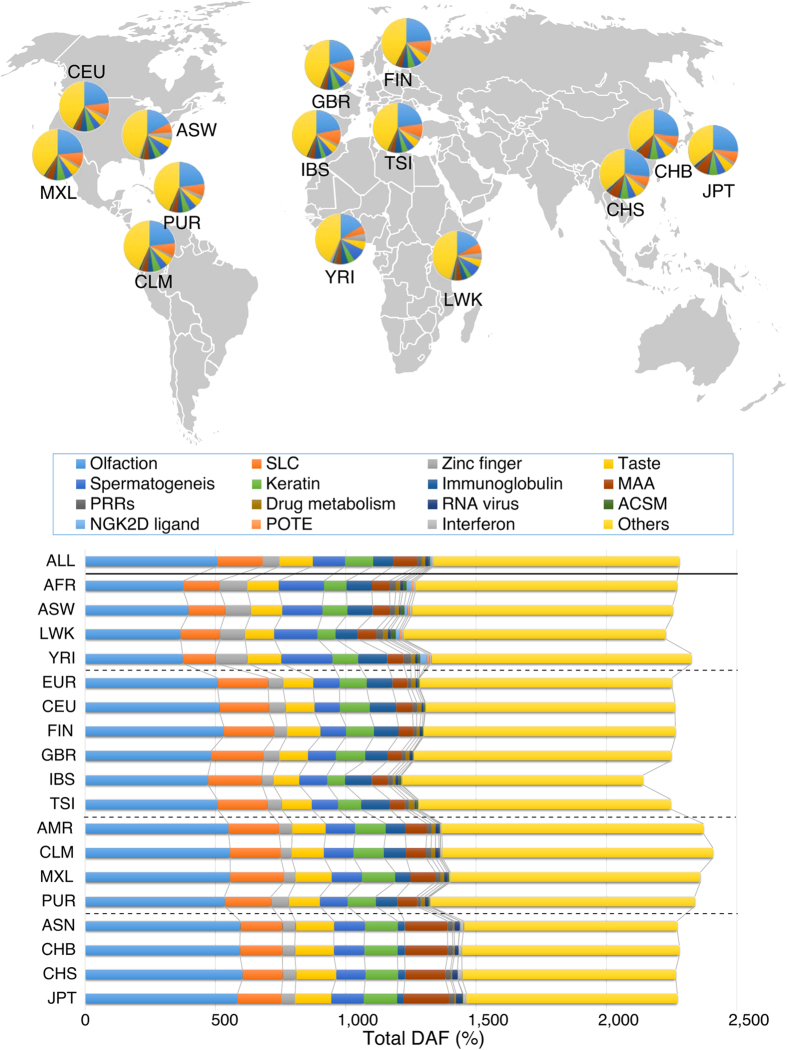
Geographical distribution of PTC mutations with DAF >1%. Geographical distributions of 235 PTC genes among 14 ethnic populations are shown in pie and bar charts. Pie areas and bar charts are constructed based on GO and STING analysis as shown in Fig. 2. A world map was obtained from Free Editable Worldmap (http://free-editable-worldmap-for-powerpoint.en.softonic.com/) and modified.

The geographical and ethnic distribution of the 15 categories of PTC genes with a DAF >1% were evaluated in 14 populations (Fig. 5). While the difference in the total DAF of PTC mutations among populations was small (22.78 ± 0.65%), their compositions were different (Fig. 5). For example, a higher total PTC DAF was observed for olfaction and keratin genes in the Asian population (olfaction 595%; keratin 127%; melanoma-associated antigen 164%) compared with Africans (olfaction 377%; keratin 86%; melanoma-associated antigen 67%), Europeans (olfaction 509%; keratin 104%; melanoma-associated antigen 58%), and Americans (olfaction 551%; keratin 118%; melanoma-associated antigen 83%) (Fig. 5). In contrast, PTC DAF was higher for zinc finger and spermatogenesis genes in Africans than in Asians (Fig. 5). PTCs were more frequent in solute carrier (SLC) genes in Europeans and Americans compared with Asians, and lowest in Africans (Fig. 5).

## Discussion

Knowing the types of genes or biological processes that are evolving in modern humans is one of the key objectives of population genetics. There are hidden records in the DNA of modern humans that can help point us to the traits that have become detrimental or beneficial for human survival. A number of approaches have been used to identify evolutionarily significant variations involved in adaptation^3^,^4^,^5^,^6^,^7^,^8^,^9^. The traditional one is re-sequencing the candidate genes that likely played a crucial role in adaptation from a panel of ethnically diverse humans and other primates^3^,^4^,^5^,^6^,^7^. More recently, genome-wide approaches using diverse populations have been used to identify candidate genomic regions that have been targets of natural selection during human history^8^,^9^. The research here was aimed at finding loci where there was a strong recent selection of alleles that had not yet reached fixation, using the two largest population exomes. Here, 246 high-frequency PTC mutations in 231 genes were analyzed as a representation of recent genomic alternations. Note that the allele frequency has not changed rapidly at the one-generation level.

By integrating the GO and interactome data of 246 PTC genes, the results showed that recent PTC mutations during modern human evolution were limited to 15 specific pathways: olfaction, solute carriers, zinc fingers, taste, spermatogenesis, keratin, immunoglobulin, melanoma-associated antigens, drug metabolism, endogenous RNA virus element, medium-chain Acyl-CoA synthetase, ligands for NGK2D, POTE ankyrin domain, interferons, and immune defense receptors (Fig. 2). Several categories, including chemosensory perception and gametogenesis, have been identified as targets of natural selection in previous studies of human-chimpanzee divergence^38^,^39^ while seven other categories were previously unreported (see also Supplementary Notes). Combination of GO and interactome analysis has the potential to unveil evolutionary trends in modern humans.

A note of caution must be added about potential sampling biases introduced in the analysis due to using only two datasets, NHLBI and 1000G. NHLBI contains individuals sequenced as part of various disease-specific studies and may not reflect the precise genetic population structures, although no previous reports showed any relationship between the NHLBI target diseases and PTC mutations. The 1000G project collected healthy individuals, and in theory is more representative of population diversity surveyed, although the sample size was limited (around 100 individuals per population). Furthermore it is also necessary to note that exome sequencing occasionally cannot cover full exons due to technical limitations, and unsequenced PTCs are indistinguishable from absent ones.

### Selective forces on PTC mutations

Although PTC mutations are common causes of genetic disorders, some variants have proven to be advantageous in recent human evolution^40^,^41^,^42^,^43^,^44^. The most famous examples of beneficial variants are the PTC alleles in the *CASP12* (OMIM *608633; rs497116; p.Arg125*) and *ACTN3* (OMIM+102574; rs1815739; p.Arg577*) genes. Here, these variants were confirmed to be present at high frequencies (*CASP12*, DAF = 96.0%; *ACTN3*, DAF = 36.1% (1000G)) and frequently occurred in a homozygous state (Table S1). Carriers of the PTC mutation in *CASP12* are more resistant to infection and severe sepsis^40^,^41^, and the PTC allele in *ACTN3* has been associated with increased endurance in athletic performance^42^,^43^. In addition, the PTC mutation in *TLR5* (OMIM *603031; rs5744168, p.Arg392*) (DAF = 4.0% (1000G); Table S1) is associated with resistance to autoimmune disease and obesity^44^. These previous studies have provided us with important insights into the functional elements within the genome and the effects of PTC mutations. Loss of NGK2D ligands, interferons, and immune defense receptors may protect us from hypersensitive immune reactions, although homozygous humans deficient in these genes may have gradually increased the susceptibility to pathogens (see Supplementary Notes). The reduction of the functional Cytochrome P450 repertoire may be associated with decreased risks of daily exposure to toxicants and modern humans may have been losing this drug-metabolizing ability. The true effects of PTC mutations in melanoma-associated antigens, medium-chain Acyl-CoA synthetase, and POTE ankyrin domain are still unknown, but the PTCs have a strong tendency to be introduced into members of the same gene family, even during modern human evolution. Closely related genes may compensate for the function of lost genes. The unclustered genes described in Fig. 2B, including *CASP12, ACTN3* and *TLR3*, may also play an interesting role in human evolutionary history.

Recently, researchers have begun to propose a new project for analyzing human genetic traits, which was discussed at the American Society of Human Genetics annual meeting 2014^45^. The method is similar to knockout animal models, in which a target gene is inactivated to determine its function, except that instead of animals, the method is non-invasive and focuses on human subjects^45^. Each individual carries about 200 loss-of-function variants^45^. If large sample sizes are available, this strategy is especially useful for identifying harmful or beneficial mutations that might contribute to human health^45^. Studying knockout mutations in modern humans has enormous potential to not only improve diagnosis and treatment of many diseases, but also to decipher human evolution. The identified loci are candidate genes involved in recent adaptations or vulnerabilities of modern humans. They could provide details on ways modern humans may have adapted to selective pressures in the most recent stage of our history. Further genetic studies are needed to uncover the total effect of each PTC mutation on individual lives and to fundamentally understand the evolution of modern humans.

## Materials and Methods

### Descriptive statistics of PTC variants from exome sequences

Most calculations and data reductions in following procedures were performed using macro functions (VBA) in Excel. Data were collected from the genotyping pipeline from NHLBI (http://www.nhlbi.nih.gov/)^21^ and 1000 Genomes (http://www.ncbi.nlm.nih.gov/variation/tools/1000genomes/)^22^, which consisted of high-coverage exome sequence data from various ethnic groups. The datasets (VCF files) were filtered on Variant Tools (http://varianttools.sourceforge.net/Annotation/HomePage) and Microsoft Excel by total read depth, the number of individuals with coverage at the site and the average position of variant alleles along a read. A total of 16,281 PTC mutations were organized and classified according to mutation type, allele frequency, and ethnic group. The information about mutation types, positions and reference sequences pathogenicity were retrieved from NCBI dbSNP (http://www.nlm.nih.gov/SNP/) and UCSC genome browser (http://genome.ucsc.edu/) to generate the catalogue of PTC mutations, access the validity of variants, and examine previous gene annotations. The ancestral alleles were re-determined by using four primate sequences (human, chimpanzee, [ChimpGSAC 2.1.4/panTro4), gorilla [GorillaWTSI gorGor3.1/gorGor3], and orangutan [OrangutanWUGSC 2.0.2/ponAbe2]). DAF (%) was then calculated using a formula:


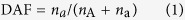


with *n*_A_ and *n*_a_ representing the number of ancestral and mutated allele, respectively.

I estimated the proportion of protein truncation each PTC would cause as the percentage of the ancestral ORF length and constructed the scatter diagram showing the ORF length-DAF-truncation percentage relationship. I could not detect only *CASP12* in NHLBI data, and 1000G data was used as necessary.

### Computational predictions of functional effects of PTC mutations

SIFT (http://sift.bii.a-star.edu.sg/)^23^ and MutationTaster (www.mutationtaster.org)^24^ were utilized to predict the effects of the PTC mutations at the protein level. SIFT incorporates phylogenetic information and the physical properties of amino acids and MutationTaster uses multiple sequence alignments and structural information for characterizing the deleterious substitution. A SIFT score <0.05 indicates that the amino acid substitution is damaging, whereas a score ≥0.05 are predicted by the algorithm to be benign. A MutationTaster prob score >0.5 indicates that the amino acid substitution is benign or deleterious in this research.

### Gene ontology (GO) analysis

GO term-enrichment analysis was performed using PANTHER 9.0 (http://www.pantherdb.org/) as previously described^26^. Inputs were the accession numbers of 235 genes containing PTC mutations with a total DAF of over 1%. All available GO terms were used, and all human genes were defined as the background. Three different modes (Molecular Function, Biological Process and Protein Class) were employed as analysis method.

### The protein-protein interaction networks

The database of protein**-**protein interactions STRING^27^ was used for the design of the weighted protein networks. The following parameters of STRING were employed: organism, required confidence (score), interactions shown were “homo sapiens”, “medium confidence (0.400)”, “no more than 20 interactions”, respectively, and the other parameters were default settings.

### PTC mutations in archaic humans

We obtained a draft of the Altai Neanderthal genome^33^ and a high-coverage Denisovan genome^34^. The BAM files were used to obtain genome sequence information. The same criteria as used in Reich *et al*.^34^ were applied to the filtering process.

### Assessment of population genetic structure

*F*_*ST*_, *G’st* and *Jost’s D* were calculated in bins with the FSTAT^46^ and SMOGD^47^ via the 14 population division. Bootstrap parameters were set to 1,000 times. I calculated their *F*_*ST*_ values to find out whether the PTC mutations were significant outliers (i.e., lying above the 95th percentiles).

## Additional Information

**How to cite this article**: Fujikura, K. Premature termination codons in modern human genomes. *Sci. Rep.* **6**, 22468; doi: 10.1038/srep22468 (2016).

## Supplementary Material

Supplementary Information
