# Supplementary material for: Premature termination codons in modern human genomes
Source: Sci Rep. 2016 Mar 2;6:22468. doi: 10.1038/srep22468 (PMC4773809; doi:10.1038/srep22468)
Supplement: Supplementary Information [file srep22468-s1.pdf]

Supplementary Information

Premature termination codons in modern human genomes

Kohei Fujikura[1]*
1 Kobe University School of Medicine, 7-5-1, Kusunoki-cho, Chuo-ku, Kobe 650-0017, Japan

***Correspondence should be addressed to Kohei Fujikura, Kobe University School of Medicine, 7-5-1, Kusunoki-cho, Chuo-ku, Kobe 650-0017, Japan.
Telephone: +81-90-3906-9772
Fax
Email: kofujikura@gmail.com

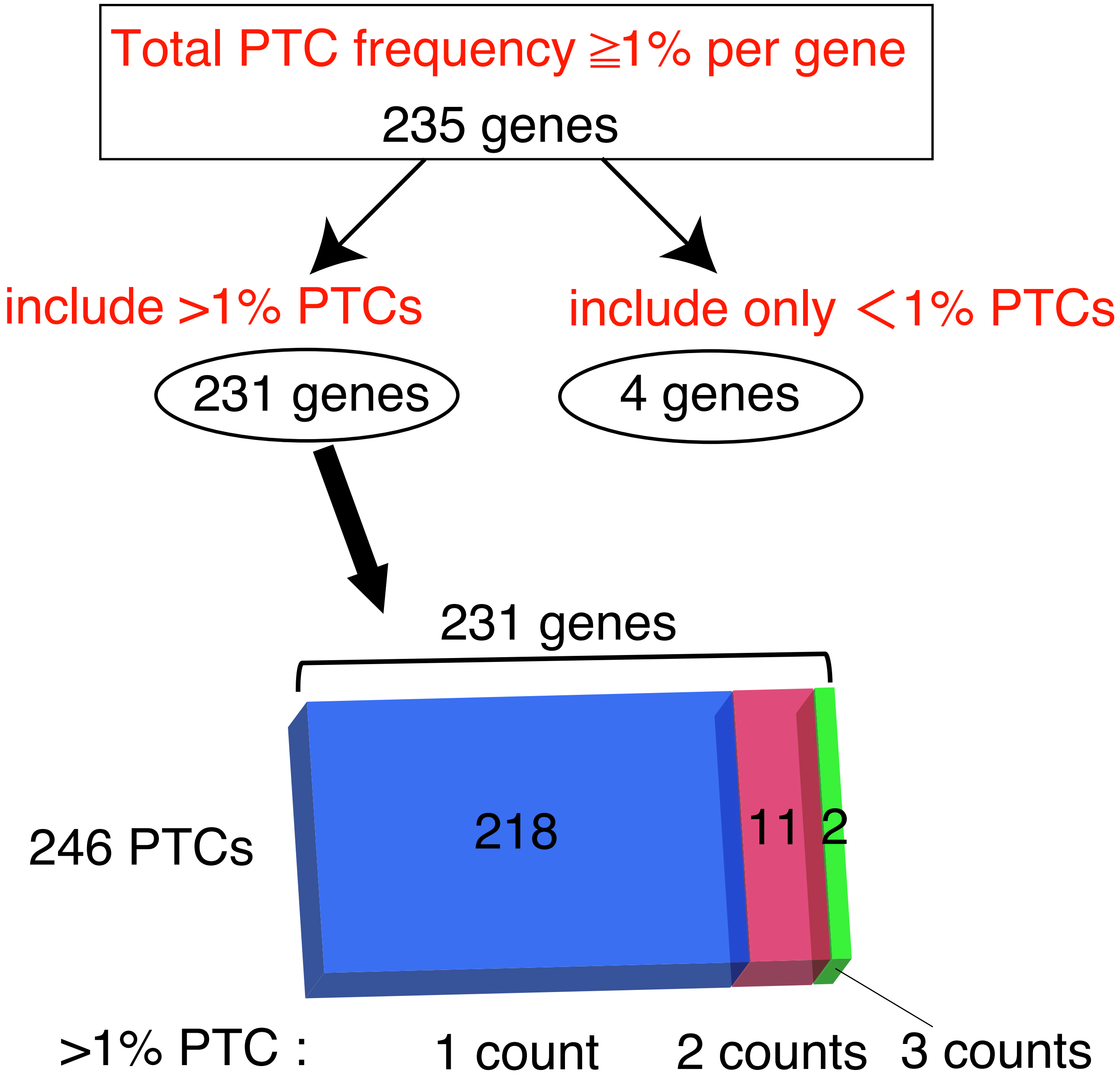
Total PTC frequency ≧1% per gene
235 genes
include >1% PTCs
231 genes
include only ＜1% PTCs
4 genes
231 genes
246 PTCs
218
11
2
>1% PTC :
1 count
2 counts
3 counts

Figure S1

Identification and classification of 246 PTC mutations with DAF >1%

A flow chart shows the classification framework of the 235 PTC genes.

Note: GO and interactome analyses in Figure 2 were performed using “235” genes. The gene functions were classified “by genes”, and thus the gene-by-gene PTC frequency was calculated and used for selection of genes. I assume that, without this criteria, it is difficult to determine which gene is frequently mutated, a gene with PTC with DAF=1.1% or a gene with two PTCs with DAF 0.9%.

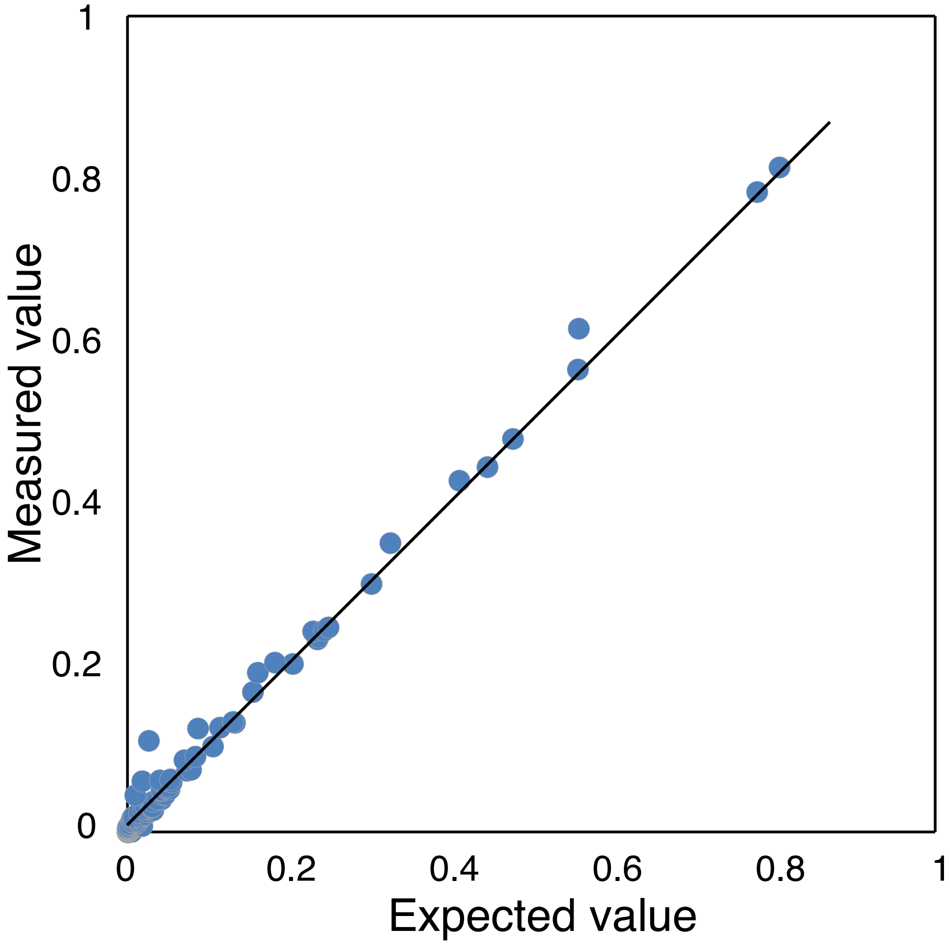
Measured value
Expected value
0
0.2
0.4
0.6
0.8
1

Figure S2

P-P plot analysis of observed vs. expected homozygosity of the PTC allele.

Even distribution across the expected line represents normal distribution (black line).

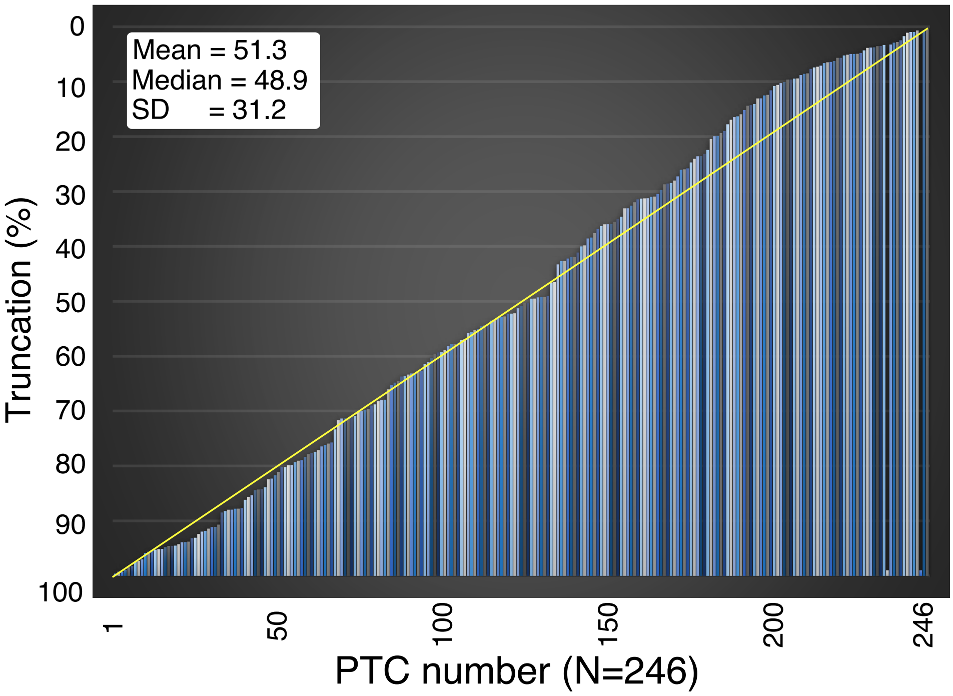
Mean = 51.3
Median = 48.9
SD = 31.2
Truncation (%)
0
10
20
30
40
50
60
70
80
90
100
1
50
100
150
200
246
PTC number (N=246)

Figure S3

Truncations as percentage of the original length of the ORF

The 246 nonsense SNPs are sorted along the x-axis by amount of protein truncation, starting at 1 for the highest truncation and ending at 246 for the lowest truncation. Even distribution of truncations of PTCs is shown in yellow. Median and average is 48.9 and 51.3%, respectively. Deviation from unity is calculated to be 1.12±2.92%.

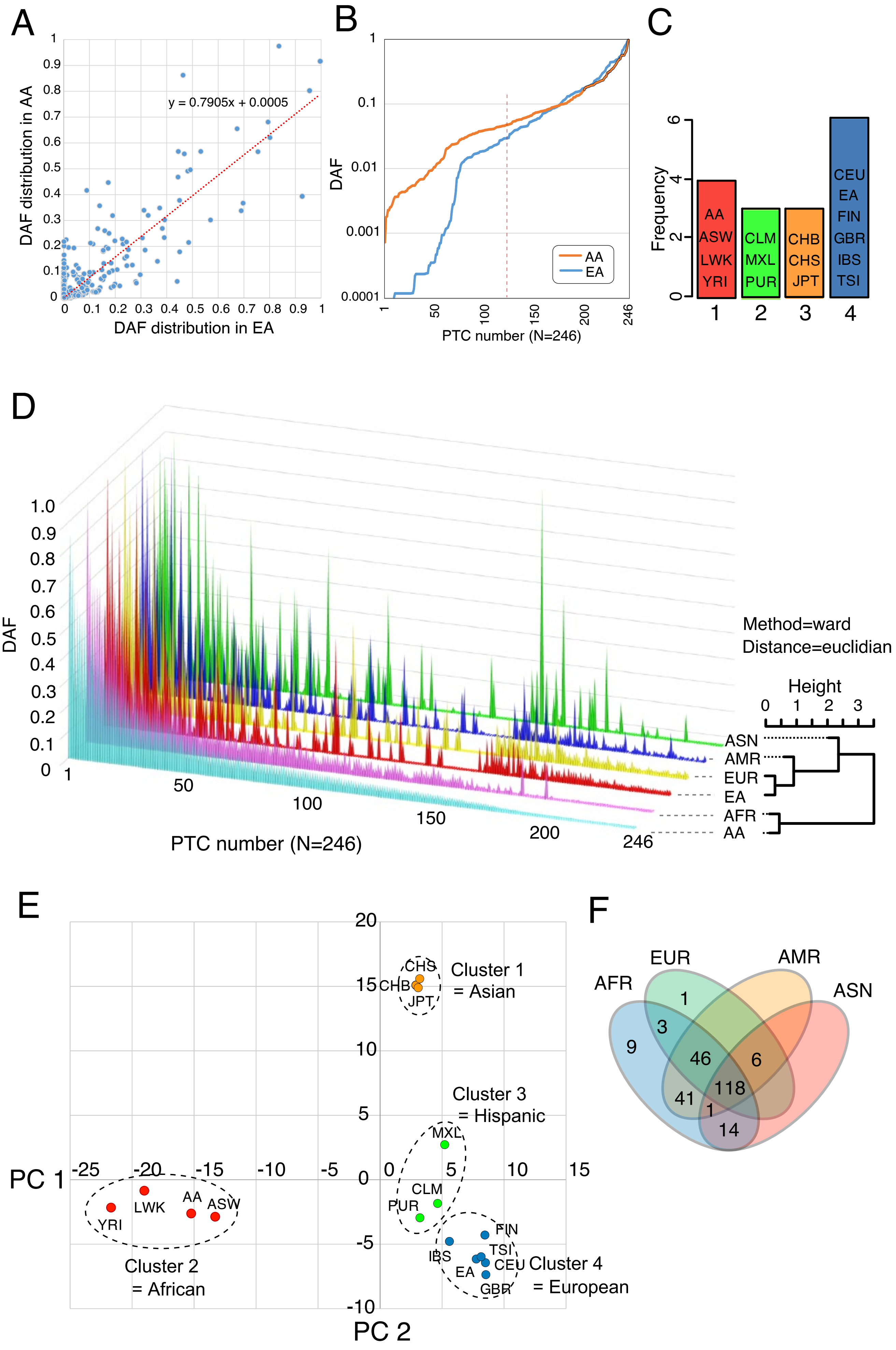
A
DAF distribution in AA
DAF distribution in EA
y = 0.7905x + 0.0005
B
DAF
PTC number (N=246)
AA
EA
C
Frequency
AA
ASW
LWK
YRI
CLM
MXL
PUR
CHB
CHS
JPT
CEU
EA
FIN
GBR
IBS
TSI
D
DAF
PTC number (N=246)
Method=ward
Distance=euclidian
Height
ASN
AMR
EUR
EA
AFR
AA
E
PC 1
PC 2
Cluster 1
= Asian
Cluster 2
= African
Cluster 3
= Hispanic
Cluster 4
= European
CHS
CHB
JPT
YRI
LWK
AA
ASW
MXL
CLM
PUR
FIN
TSI
IBS
CEU
EA
GBR
F
AFR
EUR
AMR
ASN

Figure S4

Population distribution of 246 PTCs and multivariate analysis

PTC mutation, which is often regarded as equivalent to knockout, is a visibly clear signature of gene evolution. It is important to investigate the regional and ethnic specificity of PTC mutations. I examined the population specificity of PTC mutations by analyzing distribution of 246 PTCs among a total of 16 ethnic groups. The NHLBI datasets include the exome sequences for 6,503 individuals of two ethnic origins (AA, African Americans; EA, European Americans) and 1000G datasets included 1,092 individuals of 14 ethnic origins (ASW, American's of African Ancestry in SW; CEU, Utah Residents (CEPH) with Northern and Western European ancestry ; CHB, Han Chinese in Beijing; CHS, Southern Han Chinese; CLM, Colombian from Medellin; FIN, Finnish in Finland; GBR, British in England; IBS, Iberian population in Spain; JPT, Japanese in Tokyo; LWK, Luhya in Webuye; MXL, Mexican ancestry from Los Angeles; PUR, Puerto Rico from Puerto Rica; TSI, Toscani in Italia; YRI, Yoruba in Ibadan). Using the NHLBI datasets, I demonstrated that the DAF distribution differed significantly between European and African ancestry (two-tailed Mann-Whitney U test; $p<0.002$) (Figure S4A). European groups (EAs) had more PTC variants with a higher DAF per number of individuals compared with African groups (AAs). This is because

the average DAF was almost the same between EAs (0.104) and AAs (0.101) while the EA median DAF (0.029) was almost half of the AA median DAF (0.047) (Figure S4B). These differences in DAF distribution reflect an ancestral bottleneck in non-African populations (1) and recent explosive increases in population size (2,3).

A non-hierarchical (Figure S4C) and hierarchical (Figures S4D) clustering clearly showed four subdivision of human PTC diversity. Principal component analysis (PCA) supported this result (Figure S4E). Non-African populations, especially Asians, had PTC variants with a higher DAF compared with Africans (Figure S4D). These patterns of PTC distributions are similar to synonymous and non-synonymous mutations (1,4,5,6), thus suggesting that PTC mutations have also followed gene flow observed in other types of mutations.

A Venn diagram of the PTC variants of four different populations (African, European, American and Asian) with a DAF >1% showed that approximately half (118/239) of the PTC mutations were shared between all four populations (Figure S4F). African samples had more specific variant sites (total 9) compared with non-Africans, while Europeans had only one specific variant site and Americans and Asians likely had none (Figure S4F).

References in Figure S4

1. Altshuler D, Durbin RM, Abecasis GR, et al. (2010) A map of human genome variation from population-scale sequencing. *Nature* 467: 1061-1073.

2. Coventry, A. et al. Deep resequencing reveals excess rare recent variants consistent with explosive population growth. *Nat Commun* **1**, 131 (2010).

3. Keinan, A., Clark, A.G. Recent explosive human population growth has resulted in an excess of rare genetic variants. *Science* **336**, 740-743. (2012).

4. Altshuler, D. *et al*. A map of human genome variation from population-scale sequencing. *Nature* **467**, 1061-1073 (2010).

5. Abraham, G. & Inouye, M. Fast principal component analysis of large-scale genome-wide data. *PLoS One* **9**, e93766 (2014).

6. Qin, P. *et al*. Quantitating and dating recent gene flow between European and East Asian populations. *Sci Rep* **5**, 9500 (2015).

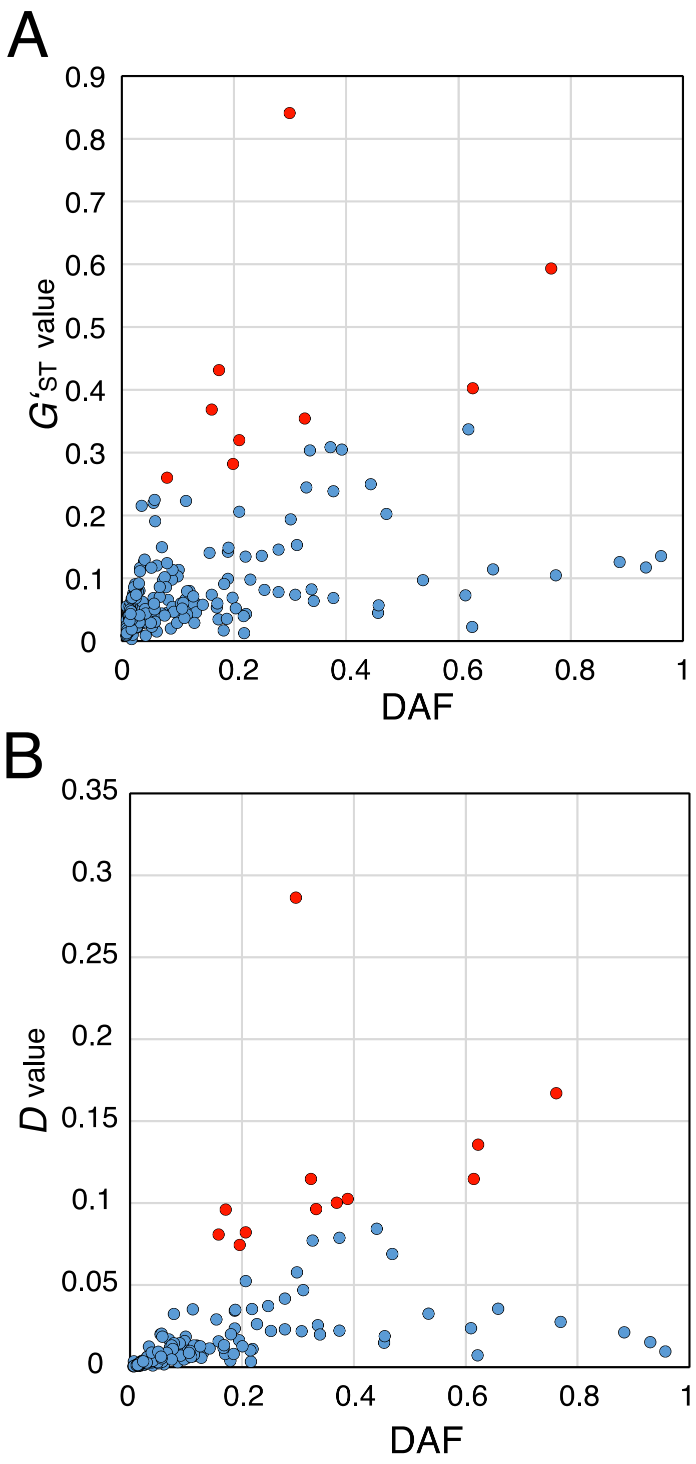
A
0.9
0.8
0.7
0.6
0.5
0.4
0.3
0.2
0.1
0
G'ST value
0
0.2
0.4
0.6
0.8
1
DAF
B
0.35
0.3
0.25
0.2
0.15
0.1
0.05
0
D value
0
0.2
0.4
0.6
0.8
1
DAF

Figure S5

G_{ST} and Jost's *D* versus DAF of 246 PTCs

The G_{ST} (A) and Jost's *D* (B) values of 246 PTCs are calculated and plotted against their DAF. Three outliers (higher than 99th percentiles) of G_{ST} are labelled in red.

Chromosomal positi	dbSNP ID	Gene Name	Accession numb	NHLBI (derived allele frequency) Total	AA	EA	1000 Genomes (derived allele frequency) ALL	AFR	AMR	ASN	EUR	ASW	CEU	CHB	CHS	CLM	FIN	GBR	IBS	JPT	LWK	MXL	PUR	TSI	YRI	Amino Acid change	Truncated/Full Protein (	Trancation (%	Heterozygosit	F_{ST}	G_{ST}	Jost's D
Chr4:130030763	rs10009430	C4orf33	NM 001099783.1	0.0213	0.0615	0.0007	0.015	0.061	0.006	0	0	0.082	0	0	0	0.008	0	0	0	0	0.062	0	0.009	0	0.045	p.Arg144*	144/200	72.0	0.0293	0.0014	0.0015	0.0014
Chr2:85921552	rs10181739	GNLY	NM 006433.3	0.0275	0.0810	0.0001	0.025	0.104	0.011	0	0	0.115	0	0	0	0	0	0	0	0	0.113	0	0.036	0	0.085	p.Trp4*	4/146	2.7	0.0467	0.0759	0.0830	0.0039
Chr7:63529269	rs10237332	ZNF727	NM 001159522.1	0.0114	0.0376	0.0000	0.011	0.041	0.006	0.002	0	0.033	0	0	0	0.008	0	0	0	0.006	0.036	0.008	0	0	0.051	p.Arg2*	2/500	0.4	0.0211	0.0265	0.0276	0.0005
Chr5:41061715	rs1023840	HEATR7B2	NM 173489.4	0.1530	0.0543	0.1992	0.154	0.02	0.268	0.248	0.116	0.041	0.112	0.247	0.23	0.292	0.081	0.174	0	0.27	0.01	0.295	0.209	0.117	0.017	p.Trp191*	191/1586	12.0	0.2207	0.0867	0.1392	0.0288
Chr7:149528262	rs10261977	SSPO	NM 198455.2	0.1746	0.1961	0.1647	0.179	0.189	0.21	0.192	0.146	0.164	0.147	0.247	0.14	0.225	0.134	0.146	0.143	0.191	0.211	0.22	0.182	0.158	0.182	p.Arg5060*	5060/5148	98.3	0.3022	0.0084	0.0153	0.0035
Chr19:54140168	rs10409173	DPRX	NM 001012728.1	0.0151	0.0445	0.0001	0.010	0.041	0.006	0	0	0.033	0	0	0	0	0	0	0	0	0.052	0.008	0.009	0	0.034	p.Glu168*	168/192	87.5	0.0201	0.0279	0.0289	0.0005
Chr19:49445774	rs10423255	DHDH	NM 014475.3	0.0595	0.0592	0.0597	0.060	0.057	0.055	0.061	0.062	0.033	0.1	0.052	0.045	0.058	0.043	0.045	0	0.09	0.077	0.061	0.045	0.071	0.051	p.Gln233*	233/335	69.6	0.1062	0.0110	0.0136	0.0013
Chr17:74077797	rs1043149	ZACN	NM 180990.3	0.1500	0.0990	0.1762	0.218	0.063	0.307	0.33	0.193	0.098	0.106	0.361	0.345	0.292	0.204	0.197	0.321	0.281	0.036	0.379	0.236	0.235	0.068	p.Gln281*	281/413	68.0	0.2903	0.0676	0.1329	0.0350
Chr3:53899276	rs1043261	IL17RB	NM 018725.3	0.0840	0.0937	0.0790	0.114	0.136	0.077	0.177	0.07	0.107	0.065	0.186	0.23	0.083	0.054	0.079	0.071	0.107	0.17	0.076	0.073	0.082	0.119	p.Gln484*	484/503	96.2	0.1987	0.0265	0.0386	0.0062
Chr10:1065710	rs1044261	IDI2	NM 033261.2	0.0667	0.0474	0.0765	0.041	0.037	0.052	0	0.069	0.057	0.076	0	0	0.075	0.054	0.09	0.107	0	0.031	0.008	0.082	0.051	0.028	p.Trp144*	144/228	63.2	0.0797	0.0275	0.0328	0.0027
Chr5:68616079	rs10471773	CCDC125	NM 176816.3	0.0160	0.0054	0.0214	0.016	0	0.022	0	0.037	0	0.024	0	0	0.05	0.043	0.034	0.071	0	0	0.008	0.009	0.041	0	p.Arg97*	97/512	18.9	0.0311	0.0270	0.0291	0.0011
Chr17:67149973	rs10491178	ABCA10	NM 080282.3	0.0600	0.0738	0.0530	0.100	0.087	0.157	0.122	0.065	0.082	0.041	0.165	0.125	0.142	0.118	0.051	0	0.073	0.108	0.174	0.155	0.056	0.068	p.Arg1322*	1322/1544	85.6	0.1731	0.0287	0.0405	0.0061
Chr11:48286231	rs10838851	OR4X1	NM 001004726.1	0.6906	0.5659	0.7545	0.660	0.528	0.738	0.601	0.753	0.508	0.706	0.629	0.55	0.758	0.828	0.787	0.679	0.629	0.474	0.742	0.709	0.704	0.602	p.Tyr273*	273/306	89.2	0.4359	0.0455	0.1130	0.0353
Chr1:248113026	rs10888281	OR2L8	NM 001001963.1	0.7477	0.3951	0.9283	0.764	0.291	0.934	0.832	0.939	0.393	0.941	0.814	0.845	0.925	0.957	0.933	1	0.837	0.268	0.962	0.909	0.918	0.244	p.Tyr289*	289/313	92.3	0.2372	0.3880	0.5926	0.1673
Chr9:115759519	rs10981589	ZNF883	NM 001101338.1	0.0332	0.0084	0.0456	0.018	0	0.017	0.003	0.041	0	0.065	0.01	0	0.017	0.027	0.045	0	0	0	0.023	0.009	0.036	0	p.Arg341*	341/380	89.7	0.0339	0.0237	0.0252	0.0008
Chr15:68497597	rs11071990	CALML4	NM 001031733.2	0.0824	0.2255	0.0092	0.069	0.226	0.011	0.051	0.008	0.23	0.006	0.067	0.05	0	0	0.022	0.036	0.034	0.216	0.015	0.018	0	0.233	p.Arg40*	40/150	26.7	0.1227	0.1189	0.1480	0.0165
Chr22:36556768	rs11089781	APOL3	NM 145640.2	0.0744	0.2183	0.0006	0.054	0.232	0.014	0	0	0.156	0	0	0	0.017	0	0	0	0	0.211	0	0.027	0	0.307	p.Gln58*	58/403	14.4	0.0778	0.1869	0.2190	0.0198
Chr19:57646393	rs111350153	ZIM3	NM 052882.1	0.0156	0.0050	0.0210	0.009	0.004	0	0	0.022	0.016	0.047	0	0	0	0.016	0.011	0	0	0	0	0	0.02	0	p.Lys438*	438/473	92.6	0.0174	0.0218	0.0225	0.0003
Chr4:70512787	rs111696697	UGT2A1	NM 006798.2	0.0304	0.0091	0.0413	0.019	0.004	0.022	0.002	0.04	0.016	0.029	0	0.005	0.025	0.027	0.039	0.036	0	0	0.015	0.027	0.061	0	p.Tyr192*	192/528	36.4	0.0357	0.0159	0.0172	0.0007
Chr4:84206004	rs112033303	COQ2	NM 015697.7	0.0168	0.0042	0.0229	0.012	0.004	0.028	0	0.018	0.016	0.024	0	0	0.033	0.011	0.011	0	0	0	0.015	0.036	0.031	0	p.Arg22*	22/422	5.2	0.0238	0.0138	0.0145	0.0004
Chr2:108863758	rs112050262	SULT1C3	NM 001008743.1	0.0154	0.0120	0.0171	0.016	0.004	0.017	0.007	0.03		0.006	0.01	0.005	0.017	0.054	0.028		0.006	0.005	0.015	0.018	0.036	0.006	p.Trp36*	36/305	11.8	0.0321	0.0150	0.0159	0.0004
Chr11:56431216	rs11228710	OR5AR1	NM 001004730.1	0.5703	0.3378	0.6895	0.624	0.242	0.751	0.799	0.679	0.361	0.724	0.778	0.815	0.717	0.683	0.624	0.643	0.803	0.191	0.795	0.736	0.694	0.216	p.Gln19*	19/311	6.1	0.3855	0.1787	0.4017	0.1358
Chr11:62848487	rs11231341	SLC22A24	NM 001136506.2	0.7466	0.6207	0.8014	0.772	0.606	0.818	0.841	0.806	0.631	0.788	0.84	0.865	0.808	0.828	0.82	0.821	0.815	0.639	0.894	0.736	0.786	0.551	p.Tyr501*	501/553	90.6	0.3297	0.0518	0.1034	0.0272
Chr6:32632638	rs1130385	HLA-DOB1	NM 002123.4	0.0104	0.0139	0.0087	0.170	0.187	0.18	0.133	0.182	0.221	0.171	0.144	0.135	0.175	0.215	0.14	0.321	0.118	0.124	0.182	0.182	0.179	0.233	p.Glu106*	106/262	40.5	0.1749	0.0181	0.0331	0.0076
Chr12:11243929	rs113710768	TAS2R43	NM 176884.2	0.1627	0.1057	0.1891	No data	No data	No data	No data	No data	No data	No data	No data	No data	No data	No data	No data	No data	No data	No data	No data	No data	No data	No data	p.Trp300*	300/310	96.8	No data	No data	No data	No data
Chr14:73965778	rs114232158	HEATR4	NM 001220484.1	0.0108	0.0318	0.0000	0.011	0.047	0	0	0	0.049	0	0	0	0	0	0	0	0	0.026	0	0	0	0.068	p.Arg785*	785/1027	76.4	0.0211	0.0442	0.0460	0.0009
Chr21:43531325	rs114358105	UMODL1	NM 001199527.1	0.0162	0.0235	0.0128	0.016	0.037	0.019	0	0.012	0.049	0.012	0	0	0.025	0.016	0.022	0	0	0.031	0.023	0.009	0	0.034	p.Gln593*	593/1375	43.1	0.0293	0.0145	0.0154	0.0005
Chr1:35227093	rs114429815	GJB4	NM 153212.2	0.0115	0.0336	0.0001	0.009	0.033	0.008	0	0	0.025	0	0	0	0.008	0	0	0	0	0.036	0	0.018	0	0.034	p.Gln80*	80/267	30.0	0.0174	0.0201	0.0208	0.0004
Chr3:52005638	rs114730569	ABHD14B	NM 001146314.1	0.0157	0.0458	0.0002	0.011	0.049	0	0	0	0.025	0	0	0	0	0	0	0	0	0.046	0	0	0	0.068	p.Gln17*	17/211	8.1	0.0201	0.0437	0.0454	0.0009
Chr10:128153385	rs114882604	C10orf90	NM 001004298.2	0.0129	0.0379	0.0001	0.011	0.039	0.017	0	0	0.033	0	0	0	0	0	0	0	0	0.046	0	0.055	0	0.034	p.Arg472*	472/700	67.4	0.0229	0.0323	0.0338	0.0008
Chr16:82033810	rs11542462	SDR42E1	NM 145168.2	0.0920	0.0225	0.1242	0.071	0.008	0.08	0.002	0.16	0.008	0.159	0.005	0	0.075	0.113	0.129	0.143	0	0.015	0.076	0.091	0.235	0	p.Gln30*	30/394	7.6	0.1218	0.0737	0.0954	0.0117
Chr12:56077768	rs115687886	METTL7B	NM 152637.2	0.0137	0.0397	0.0003	0.011	0.043	0.008	0	0	0.033	0	0	0	0.008	0	0	0	0	0.046	0	0.018	0	0.045	p.Arg224*	224/245	91.4	0.0201	0.0272	0.0283	0.0006
Chr3:9874914	rs115917139	TTLL3	NM 001025930.3	0.0322	0.0073	0.0438	0.020	0	0.03	0	0.042	0	0.065	0	0	0.05	0.027	0.045	0	0	0	0.015	0.027	0.041	0	p.Arg704*	704/916	76.9	0.0394	0.0260	0.0280	0.0010
Chr6:155577717	rs116268446	TIAM2	NM 001010927.2	0.0168	0.0411	0.0043	0.010	0.026	0.014	0	0.005	0.008	0.006	0	0	0.008	0	0	0.036	0	0.01	0	0.036	0.01	0.057	p.Leu448*	448/627	71.5	0.0201	0.0242	0.0253	0.0006
Chr1:109823457	rs116389032	PSRC1	NM 001005290.3	0.0175	0.0041	0.0243	0.008	0	0.019	0	0.015	0	0.024	0	0	0.017	0.011	0.017	0	0	0	0.015	0.027	0.01	0	p.Lys249*	249/311	80.1	0.0165	0.0107	0.0110	0.0002
Chr10:37419292	rs116939015	ANKRD30A	NM 052997.2	0.0137	0.0037	0.0183	0.010	0	0.014	0	0.021	0	0.024	0	0	0.017	0.005	0.039	0.036	0	0	0.008	0.018	0.015	0	p.Glu110*	110/1342	8.2	0.0174	0.0153	0.0160	0.0004
Chr11:56086560	rs117366703	OR8K3	NM 001005202.1	0.0112	0.0036	0.0151	0.010	0	0.011	0	0.022	0	0.018	0	0	0.025	0.043	0.011	0	0	0	0	0.009	0.02	0	p.Gln260*	260/313	83.1	0.0192	0.0181	0.0188	0.0003
Chr8:52284560	rs117752382	PXDNL	NM 144651.4	0.0130	0.0018	0.0184	0.012	0.004	0.025	0	0.02	0.016	0.012	0	0	0.042	0.011	0.017	0	0	0	0.008	0.027	0.041	0	p.Cys1258*	1258/1464	85.9	0.0220	0.0168	0.0176	0.0004
Chr17:45468858	rs118004742	C17orf57	NM 001195192.1	0.0443	0.0148	0.0594	0.029	0.004	0.08	0	0.044	0	0.035	0	0	0.025	0.043	0.051	0	0	0.01	0.045	0.182	0.051	0	p.Tyr450*	450/785	57.3	0.0549	0.0703	0.0788	0.0046
Chr22:18912677	rs11913840	PRODH	NM 001195226.1	0.0583	0.1010	0.0364	0.046	0.102	0.039	0.023	0.03	0.066	0.029	0.031	0.03	0.033	0.032	0.028	0.036	0.006	0.108	0.023	0.064	0.031	0.119	p.Trp77*	77/493	15.6	0.0842	0.0229	0.0271	0.0022
Chr1:40773150	rs12077871	COL9A2	NM 001852.3	0.0314	0.0906	0.0010	0.051	0.096	0.025	0.094	0.003	0.057	0	0.113	0.09	0.025	0.011	0	0	0.079	0.113	0.03	0.018	0	0.102	p.Gln326*	326/690	47.2	0.0916	0.0435	0.0514	0.0041
Chr1:20501582	rs12139100	PLA2G2C	NM 001105572.1	0.1997	0.2504	0.1760	0.185	0.242	0.124	0.164	0.191	0.221	0.206	0.144	0.215	0.117	0.145	0.23	0.107	0.129	0.247	0.091	0.173	0.199	0.25	p.Arg36*	36/151	23.8	0.3068	0.0187	0.0337	0.0076
Chr10:4889403	rs12240276	AKR1E2	NM 001040177.1	0.1096	0.0990	0.1151	0.077	0.093	0.086	0	0.121	0.074	0.106	0	0	0.125	0.145	0.101	0.107	0	0.072	0.068	0.064	0.133	0.131	p.Arg301*	301/321	93.8	0.1328	0.0321	0.0428	0.0055
Chr19:37310351	rs1227794	ZNF790	NM 001242800.1	0.0285	0.0840	0.0001	0.020	0.081	0.011	0	0	0.074	0	0	0	0.008	0	0	0	0	0.077	0.008	0.018	0	0.091	p.Arg299*	299/637	46.9	0.0366	0.0543	0.0584	0.0022
Chr1:159410340	rs12409540	OR10J1	NM 012351.2	0.1071	0.0406	0.1412	0.080	0.016	0.152	0.019	0.133	0.033	0.147	0.015	0.025	0.158	0.129	0.135	0.143	0.017	0	0.159	0.136	0.122	0.023	p.Cys264*	264/321	82.2	0.1383	0.0470	0.0641	0.0090
Chr22:29837884	rs12484086	RFPL1	NM 021026.2	0.1791	0.1918	0.1726	0.129	0.199	0.108	0.03	0.169	0.18	0.188	0.036	0.02	0.133	0.177	0.185	0.071	0.034	0.201	0.091	0.1	0.143	0.21	p.Gln243*	243/318	76.4	0.2179	0.0380	0.0585	0.0107
Chr5:134782450	rs12520799	C5orf20	NM 130848.2	0.4015	0.1378	0.5366	0.390	0.081	0.492	0.448	0.499	0.123	0.488	0.407	0.4	0.475	0.608	0.483	0.5	0.545	0.098	0.508	0.491	0.418	0.034	p.Arg117*	117/245	47.8	0.4176	0.1241	0.3038	0.1026
Chr1:152323132	rs12568784	FLG2	NM 001014342.2	0.1934	0.2606	0.1591	0.299	0.293	0.34	0.486	0.142	0.336	0.141	0.505	0.48	0.383	0.172	0.124	0.107	0.472	0.284	0.356	0.273	0.138	0.273	p.Ser2377*	2377/2392	99.4	0.3837	0.0875	0.1925	0.0575
Chr12:11461802	rs12829245	PRB4	NM 002723.3	0.1353	0.0405	0.1838	0.099	0.018	0.177	0.003	0.187	0.057	0.218	0.01	0	0.175	0.177	0.219	0.107	0	0.01	0.152	0.209	0.153	0	p.Arg39*	39/248	15.7	0.1804	0.0785	0.1118	0.0181
Chr16:81199544	rs12925771	PKD1L2	NM 052892.3	0.2299	0.1869	0.2508	0.370	0.199	0.362	0.643	0.277	0.238	0.271	0.675	0.65	0.358	0.333	0.292	0.107	0.601	0.196	0.455	0.255	0.24	0.176	p.Arg1040*	1040/2460	42.3	0.3745	0.1346	0.3079	0.1002
Chr19:43373034	rs12986075	PSG1	NM 001184825.1	0.1410	0.0456	0.1939	0.097	0.01	0.124	0.021	0.197	0.041	0.206	0.021	0.025	0.175	0.183	0.197	0.107	0.017	0	0.068	0.136	0.214	0	p.Arg288*	288/420	68.6	0.1474	0.0725	0.1014	0.0156
Chr16:55903554	rs13338754	CES5A	NM 001143685.1	0.0318	0.0921	0.0009	0.026	0.104	0.014	0	0	0.074	0	0	0	0.008	0	0	0	0	0.108	0.008	0.027	0	0.119	p.Gln174*	174/576	30.2	0.0476	0.0708	0.0774	0.0036
ChrX:75004529	rs1343879	MAGEE2	NM 138703.4	0.0290	0.0289	0.0290	0.297	0.045	0.245	0.907	0.034	0.112	0.032	0.92	0.833	0.22	0.086	0.022	0	0.977	0.034	0.356	0.134	0	0.008	p.Glu120*	120/524	22.9	X chromosome	0.6262	0.8407	0.2870
Chr17:39254247	rs137943557	KRTAP4-8	NM 031960.2	0.0333	0.0079	0.0443	0.018	0	0.017	0	0.045	0	0.065	0	0	0.033	0.032	0.039	0	0	0	0.015	0	0.051	0	p.Cys30*	30/186	16.1	0.0330	0.0291	0.0310	0.0010
Chr8:143763531	rs138377917	PSCA	NM 005672.4	0.0250	0.0069	0.0341	0.012	0.004	0.017	0	0.025	0.008	0.018	0	0	0.017	0.005	0.051	0.071	0	0.005	0.008	0.027	0.02	0	p.Trp109*	109/115	94.8	0.0247	0.0256	0.0273	0.0009
Chr22:17072347	rs139948519	CCT8L2	NM 014406.4	0.0253	0.0683	0.0033	0.013	0.053	0.003	0	0.003	0.041	0	0	0	0.008	0	0	0.071	0	0.046	0	0	0	0.068	p.Trp365*	365/558	65.4	0.0266	0.0419	0.0447	0.0014
Chr3:97851762	rs140171913	OR5H1	NM 001005338.1	0.0123	0.0356	0.0002	0.011	0.049	0.003	0	0	0.008	0	0	0	0	0	0	0	0	0.052	0	0.009	0	0.074	p.Ser74*	74/314	23.6	0.0211	0.0482	0.0501	0.0010
Chr1:104297389	rs140363602	AMY1C	NM 001008219.1	0.0103	0.0025	0.0143	No data	No data	No data	No data	No data	No data	No data	No data	No data	No data	No data	No data	No data	No data	No data	No data	No data	No data	No data	p.Arg352*	352/512	68.8	No data	No data	No data	No data
Chr7:64438667	rs1404453	ZNF117	NM 015852.3	0.8985	0.7834	0.9554	0.886	0.728	0.945	0.885	0.962	0.713	0.971	0.861	0.91	0.933	0.952	0.955	0.929	0.882	0.768	0.977	0.918	0.974	0.693	p.Arg428*	428/484	88.4	0.1896	0.0861	0.1243	0.0209
Chr1:145273366	rs140871032	NOTCH2NL	NM 203458.3	0.0103	0.0068	0.0121	No data	No data	No data	No data	No data	No data	No data	No data	No data	No data	No data	No data	No data	No data	No data	No data	No data	No data	No data	p.Arg74*	74/237	31.2	No data	No data	No data	No data
Chr1:25629818	rs141833592	RHD	NM 001127691.1	0.0147	0.0398	0.0001	No data	No data	No data	No data	No data	No data	No data	No data	No data	No data	No data	No data	No data	No data	No data	No data	No data	No data	No data	p.Tyr269*	269/322	83.5	No data	No data	No data	No data
Chr1:70833400	rs143899788	HHLA3	NM 001031693.2	0.0128	0.0347	0.0002	0.008	0.03	0.008	0	0	0.033	0	0	0	0.008	0	0	0	0	0.026	0.008	0.009	0	0.034	p.Gln119*	119/122	97.5	0.0147	0.0182	0.0188	0.0003
Chr19:41349786	rs145014075	CYP2A6	NM 000762.5	0.0295	0.0402	0.0240	No data	No data	No data	No data	No data	No data	No data	No data	No data	No data	No data	No data	No data	No data	No data	No data	No data	No data	No data	p.Ser467*	467/495	94.3	No data	No data	No data	No data
Chr11:55339652	rs1459101	OR4C16	NM 001004701.2	0.2194	0.1274	0.2665	0.277	0.096	0.348	0.374	0.288	0.123	0.265	0.397	0.37	0.283	0.366	0.219	0.143	0.354	0.052	0.439	0.309	0.316	0.125	p.Gln17*	17/311	5.5	0.3855	0.0669	0.1443	0.0415
Chr6:167595300	rs146018563	TCP10L2	NM 001145121.1	0.0110	0.0043	0.0138	0.008	0	0.011	0	0.018	0	0.035	0	0	0.025	0.005	0.022	0	0	0	0.008	0	0.015	0	p.Arg320*	320/354	90.4	0.0147	0.0165	0.0170	0.0003
Chr12:52711747	rs146753414	KRT83	NM 002282.3	0.0136	0.0018	0.0197	0.013	0.002	0.008	0	0.032	0.008	0.006	0	0	0.008	0.011	0.062	0	0	0	0.008	0.009	0.051	0	p.Glu201*	201/494	40.7	0.0256	0.0308	0.0323	0.0007
Chr16:71967886	rs147505098	PKD1L3	NM 181536.1	0.0175	0.0043	0.0233	0.011	0	0.006	0	0.028	0	0.018	0	0	0.008	0.048	0.028	0.036	0	0	0	0.009	0.015	0	p.Arg1586*	1586/1733	91.5	0.0211	0.0200	0.0209	0.0005
Chr9:125391241	rs1476860	OR1B1	NM 001004450.1	0.2270	0.0903	0.2970	0.333	0.079	0.412	0.57	0.281	0.123	0.282	0.531	0.62	0.417	0.269	0.32	0.25	0.556	0.082	0.508	0.291	0.26	0.045	p.Arg192*	192/319	60.2	0.3526	0.1362	0.3025	0.0963
Chr1:152681560	rs147765240	LCE4A	NM 178356.2	0.0191	0.0561	0.0001	0.017	0.069	0.011	0	0	0.008	0	0	0	0.017	0	0	0	0	0.113	0.008	0.009	0	0.062	p.Cys3*	3/100	3.0	0.0330	0.0648	0.0686	0.0020
Chr1:158576687	rs148998855	OR10Z1	NM 001004478.1	0.0131	0.0384	0.0002	0.011	0.049	0.003	0	0	0.033	0	0	0	0.008	0	0	0	0	0.057	0	0	0	0.051	p.Tyr153*	153/314	48.7	0.0229	0.0366	0.0381	0.0008
Chr15:81624929	rs150843673	TMC3	NM 001080532.1	0.0159	0.0049	0.0214	0.012	0.006	0.019	0	0.021	0.025	0.029	0	0	0.017	0.011	0.017	0.036	0	0	0	0.045	0.026	0	p.Ser1045*	1045/1101	94.9	0.0238	0.0155	0.0165	0.0005
ChrX:140969250	rs151171939	MAGEC3	NM 138702.1	0.0133	0.0344	0.0012	0.007	0.027	0	0	0.002	0.01	0	0	0	0	0	0	0	0	0.055	0	0.012	0.007	0.008	p.Gln193*	193/644	30.0	X chromosome	0.0345	0.0353	0.0004
Chr12:10271087	rs16910526	CLEC7A	NM 022570.4	0.0611	0.0263	0.0790	0.034	0.014	0.044	0	0.067	0.033	0.082	0	0	0.042	0.054	0.079	0.036	0	0.015	0.061	0.027	0.061	0	p.Tyr192*	192/202	95.0	0.0678	0.0238	0.0271	0.0017
Chr11:6913540	rs16919417	OR2D2	NM 003700.1	0.0151	0.0438	0.0003	0.011	0.045	0.003	0	0	0.008	0	0	0	0	0	0	0	0	0.036	0	0.009	0	0.08	p.Cys64*	64/309	20.7	0.0192	0.0495	0.0513	0.0009
Chr11:5462702	rs16930998	OR51I1	NM 001005288.2	0.0192	0.0111	0.0233	0.112	0.01	0.086	0.325	0.03	0.008	0.024	0.32	0.365	0.133	0.011	0.022	0.036	0.287	0.021	0.061	0.064	0.061	0	p.Gln15*	15/315	4.8	0.1584	0.1634	0.2219	0.0349
Chr19:52004903	rs16982743	SIGLEC12	NM 053003.2	0.2334	0.3187	0.1897	0.187	0.364	0.149	0.049	0.194	0.311	0.188	0.057	0.065	0.117	0.167	0.163	0.286	0.022	0.387	0.136	0.2	0.24	0.375	p.Gln29*	29/596	4.9	0.2656	0.0778	0.1408	0.0342
Chr22:29837599	rs16987628	RFPL1	NM 021026.2	0.0166	0.0488	0.0001	0.015	0.059	0.008	0	0	0.033	0	0	0	0	0	0	0	0	0.057	0	0.027	0	0.08	p.Arg148*	148/318	46.5	0.0293	0.0445	0.0469	0.0013
Chr19:9237263	rs17001893	OR7G3	NM 001001958.1	0.0206	0.0606	0.0001	0.013	0.055	0.003	0	0	0.049	0	0	0	0	0	0	0	0	0.057	0.008	0	0	0.057	p.Arg122*	122/313	39.0	0.0220	0.0407	0.0426	0.0010
Chr4:70898922	rs17147990	HTN3	NM 000200.2	0.0496	0.1455	0.0005	0.037	0.152	0.017	0	0	0.164	0	0	0	0.008	0	0	0	0	0.134	0	0.045	0	0.165	p.Tyr47*	47/52	90.4	0.0632	0.1107	0.1256	0.0084
Chr6:154360569	rs17174638	OPRM1	NM 001145279.2	0.0311	0.1019	0.0003	0.030	0.128	0.003	0	0.001	0.098	0.006	0	0	0.008	0	0	0	0	0.108	0	0	0	0.17	p.Gln57*	57/494	11.5	0.0577	0.1042	0.1148	0.0060
Chr6:29407955	rs17184009	OR10C1	NM 013941.3	0.0325	0.0778	0.0072	0.025	0.106	0.003	0	0.003	0.09	0	0	0	0.008	0.005	0	0	0	0.129	0	0	0.005	0.091	p.Gln55*	55/313	17.6	0.0412	0.0791	0.0861	0.0038
Chr18:51880889	rs17292725	STARD6	NM 139171.1	0.0286	0.0068	0.0398	0.016	0.004	0.028	0	0.032	0.008	0.024	0	0	0.017	0.022	0.051	0.107	0	0.005	0.023	0.045	0.02	0	p.Arg19*	19/221	8.6	0.0311	0.0352	0.0384	0.0017
Chr11:56310356	rs17547284	OR5M11	NM 001005245.1	0.0640	0.0278	0.0825	0.043	0.014	0.044	0	0.094	0.033	0.112	0	0	0.05	0.113	0.067	0.107	0	0	0.053	0.027	0.082	0.017	p.Tyr126*	126/306	41.2	0.0806	0.0383	0.0456	0.0038
Chr1:115236057	rs17602729	AMPD1	NM 000036.2	0.0946	0.0236	0.1310	0.054	0.006	0.083	0	0.111	0.016	0.106	0	0	0.133	0.113	0.14	0.143	0	0.005	0.053	0.064	0.082	0	p.Gln45*	45/781	5.8	0.0980	0.0541	0.0673	0.0070
Chr17:59667953	rs17610181	NACA2	NM 199290.3	0.1213	0.0747	0.1452	0.116	0.053	0.11	0.128	0.152	0.123	0.129	0.149	0.145	0.05	0.134	0.129	0.071	0.084	0.015	0.068	0.227	0.219	0.045	p.Arg197*	197/216	91.2	0.2033	0.0357	0.0530	0.0089
Chr19:53116811	rs17855778	ZNF83	NM 001105549.1	0.0195	0.0567	0.0005	0.022	0.096	0.006	0	0	0.057	0	0	0	0.008	0	0	0	0	0.134	0	0.009	0	0.08	p.Trp336*	336/517	65.0	0.0449	0.0772	0.0831	0.0032
Chr11:63057925	rs1790218	SLC22A10	NM 001039752.3	0.4794	0.3021	0.5701	0.470	0.278	0.594	0.42	0.574	0.303	0.524	0.464	0.425	0.608	0.629	0.584	0.679	0.365	0.284	0.644	0.518	0.541	0.256	p.Trp96*	96/542	17.7	0.4588	0.0740	0.2014	0.0688
Chr14:24470138	rs1811890	DHRS4L2	NM 001193635.1	0.0599	0.0391	0.0705	0.118	0.059	0.11	0.234	0.071	0.025	0.088	0.206	0.255	0.133	0.059	0.067	0.036	0.242	0.103	0.106	0.091	0.077	0.034	p.Arg97*	97/171	56.7	0.1969	0.0539	0.0781	0.0128
Chr15:78807407	rs183603441	AGPHD1	NM 001013619.2	0.0107	0.0043	0.0136	0.007	0	0.017	0	0.013	0	0.012	0	0	0.017	0.022	0.011	0	0	0	0	0.036	0.01	0	p.Tyr145*	145/374	38.8	0.0073	0.0075	0.0076	0.0001
Chr4:15482360	rs1861050	CC2D2A	NM 020785.2	0.0566	0.0980	0.0376	0.111	0.108	0.177	0.17	0.037	0.115	0.024	0.134	0.18	0.217	0.043	0.039	0.036	0.197	0.103	0.205	0.1	0.041	0.108	p.Arg88*	88/123	71.5	0.1960	0.0443	0.0647	0.0107
ChrX:151908845	rs1894360	CSAG1	NM 001102576.1	0.1306	0.0840	0.1573	0.088	0.061	0.069	0.023	0.162	0.102	0.176	0.007	0.013	0.088	0.185	0.146	0.143	0.055	0.048	0.059	0.061	0.144	0.045	p.Tyr28*	28/79	35.4	X chromosome	0.0383	0.0527	0.0075
Chr9:90538288	rs199825147	SPATA31C1	NM 001145124.1	0.0191	0.0007	0.0270	No data	No data	No data	No data	No data	No data	No data	No data	No data	No data	No data	No data	No data	No data	No data	No data	No data	No data	No data	p.Arg1156*	1156/1189	97.2	No data	No data	No data	No data
Chr17:15508676	rs200375190	POTEH	NM 001136213.1	0.0125	0.0030	0.0174	0.006	0.002	0.003	0	0.015	0.008	0.024	0	0	0	0.005	0.022	0	0	0	0.008	0	0.01	0	p.Trp34*	34/546	6.2	0.0119	0.0116	0.0118	0.0001
Chr17:15508676	rs201685025	CDRT1	NM 006382.3	0.0118	0.0361	0.0002	No data	No data	No data	No data	No data	No data	No data	No data	No data	No data	No data	No data	No data	No data	No data	No data	No data	No data	No data	p.Arg432*	432/753	57.4	No data	No data	No data	No data
Chr9:21481483	rs2039381	IFNE	NM 176891.4	0.0242	0.0660	0.0028	0.065	0.077	0.047	0.147	0.003	0.098	0	0.129	0.175	0.067	0.011	0	0	0.135	0.072	0.045	0.027	0	0.068	p.Gln71*	71/209	34.0	0.1145	0.0557	0.0688	0.0069
Chr2:228476140	rs2176186	C2orf83	NM 020161.3	0.2808	0.2324	0.3056	0.307	0.228	0.323	0.421	0.265	0.287	0.288	0.407	0.435	0.292	0.258	0.275	0.179	0.421	0.253	0.356	0.318	0.255	0.159	p.Trp141*	141/151	93.4	0.4515	0.0305	0.0723	0.0215
Chr12:54577718	rs2233919	SMUG1	NM 014311.2	0.0163	0.0477	0.0002	0.015	0.061	0.006	0	0	0.041	0	0	0	0	0	0	0	0	0.082	0	0.018	0	0.051	p.Gln3*	3/271	1.1	0.0275	0.0461	0.0486	0.0013
Chr6:167709702	rs2235197	UNC93A	NM 001143947.1	0.0713	0.0386	0.0880	0.103	0.037	0.075	0.147	0.125	0.033	0.088	0.144	0.165	0.058	0.145	0.118	0.143	0.129	0.052	0.114	0.045	0.143	0.023	p.Trp151*	151/416	36.3	0.1868	0.0246	0.0350	0.0054
Chr16:89261482	rs2270416	CDH15	NM 004933.2	0.0365	0.0302	0.0397	0.087	0.033	0.039	0.201	0.059	0.033	0.047	0.232	0.21	0.033	0.124	0.062	0	0.157	0.031	0.061	0.018	0.015	0.034	p.Tyr788*	788/815	96.7	0.1520	0.0744	0.0965	0.0119
Chr8:144522387	rs2272754	ZC3H3	NM 015117.2	0.0894	0.0499	0.1097	0.125	0.051	0.116	0.213	0.111	0.107	0.141	0.155	0.245	0.1	0.091	0.09	0.107	0.242	0.021	0.068	0.191	0.122	0.045	p.Ser880*	880/949	92.7	0.2042	0.0380	0.0580	0.0104
Chr1:236706300	rs2273865	LGALS8	NM 006499.4	0.0917	0.1938	0.0393	0.114	0.195	0.099	0.17	0.026	0.098	0.018	0.155	0.17	0.125	0.016	0.022	0.071	0.185	0.206	0.091	0.082	0.041	0.25	p.Leu212*	212/360	58.9	0.1987	0.0535	0.0777	0.0128
Chr7:21582963	rs2285943	DNAH11	NM 003777.3	0.4268	0.3780	0.4499	0.375	0.407	0.376	0.264	0.438	0.41	0.424	0.258	0.25	0.45	0.419	0.472	0.5	0.287	0.376	0.318	0.364	0.429	0.438	p.Glu34*	34/4524	0.8	0.4551	0.0247	0.0672	0.0218
Chr7:100371358	rs2293766	ZAN	NM 003386.1	0.0126	0.0236	0.0071	0.171	0.014	0.196	0.5	0.012	0	0	0.49	0.46	0.183	0.032	0.006	0.036	0.556	0.021	0.288	0.1	0.005	0.017	p.Trp1883*	1883/2813	66.9	0.1896	0.2955	0.4307	0.0960
Chr11:60265002	rs2298553	MS4A12	NM 001164470.1	0.4524	0.4682	0.4443	0.455	0.5	0.456	0.425	0.447	0.525	0.441	0.479	0.445	0.417	0.484	0.483	0.321	0.343	0.438	0.477	0.473	0.403	0.551	p.Gln71*	71/222	32.0	0.4643	0.0149	0.0433	0.0144
ChrX:103294760	rs2301384	H2BFM	NM 001164416.1	0.3144	0.0645	0.4408	0.327	0.048	0.325	0.425	0.436	0.112	0.44	0.427	0.433	0.341	0.444	0.416	0.429	0.414	0.048	0.267	0.378	0.445	0	p.Gln73*	73/155	47.1	X chromosome	0.1057	0.2435	0.0771
Chr21:46011583	rs233303	KRTAP10-6	NM 198688.2	0.2162	0.1607	0.2447	0.219	0.14	0.215	0.287	0.222	0.139	0.229	0.247	0.31	0.217	0.285	0.236	0.143	0.303	0.129	0.205	0.227	0.153	0.153	p.Cys261*	261/366	71.3	0.3361	0.0212	0.0418	0.0105
ChrX:142967468	rs237520	UBE2NL	NM 001012989.1	0.6669	0.6548	0.6738	0.623	0.605	0.588	0.626	0.65	0.561	0.656	0.633	0.653	0.527	0.636	0.686	0.619	0.586	0.603	0.584	0.659	0.63	0.639	p.Leu89*	89/154	57.8	X chromosome	0.0075	0.0208	0.0067
Chr11:5444136	rs2647574	OR51Q1	NM 001004757.2	0.3620	0.3026	0.3924	0.442	0.283	0.409	0.691	0.375	0.262	0.353	0.696	0.75	0.433	0.339	0.388	0.286	0.618	0.345	0.447	0.336	0.429	0.227	p.Arg236*	236/318	74.2	0.4267	0.0966	0.2488	0.0843
Chr12:11214145	rs2708381	TAS2R46	NM 176887.2	0.2276	0.2286	0.2271	0.216	0.211	0.202	0.219	0.223	0.254	0.259	0.216	0.24	0.158	0.253	0.208	0.179	0.197	0.175	0.227	0.218	0.184	0.222	p.Trp250*	250/310	80.6	0.3379	0.0056	0.0112	0.0028
Chr10:124214355	rs2736911	ARMS2	NM 001099667.1	0.1255	0.0709	0.1518	0.130	0.065	0.088	0.177	0.156	0.066	0.212	0.124	0.195	0.075	0.151	0.118	0.214	0.213	0.077	0.106	0.082	0.138	0.051	p.Arg38*	38/108	35.2	0.2225	0.0282	0.0441	0.0082
Chr14:64560092	rs2781377	SYNE2	NM 015180.4	0.0767	0.1146	0.0572	0.085	0.108	0.075	0.086	0.074	0.115	0.047	0.103	0.085	0.092	0.091	0.039	0.143	0.067	0.077	0.03	0.109	0.102	0.136	p.Trp4001*	4001/6886	58.1	0.1529	0.0132	0.0182	0.0025
Chr15:76016583	rs28413581	ODF3L1	NM 175881.3	0.0109	0.0323	0.0000	0.009	0.035	0.006	0	0	0.033	0	0	0	0.017	0	0	0	0	0.036	0	0	0	0.034	p.Gln15*	15/275	5.5	0.0174	0.0236	0.0244	0.0004
Chr22:17469049	rs28502153	GAB4	NM 001037814.1	0.3385	0.2338	0.3907	0.336	0.187	0.387	0.397	0.363	0.213	0.341	0.351	0.405	0.383	0.371	0.393	0.357	0.438	0.18	0.447	0.318	0.347	0.176	p.Gly163*	163/575	28.3	0.4487	0.0320	0.0809	0.0253
Chr18:658170	rs28602966	C18orf56	NM 001012716.2	0.0254	0.0212	0.0276	0.017	0.022	0.017	0	0.026	0.033	0.024	0	0	0.017	0.022	0.034	0.036	0	0.021	0.008	0.027	0.026	0.017	p.Tyr26*	26/124	21.0	0.0339	0.0080	0.0086	0.0003
Chr6:31124849	rs3130453	CCHCR1	NM 001105563.1	0.4851	0.4911	0.4817	0.456	0.502	0.519	0.355	0.472	0.541	0.488	0.366	0.38	0.55	0.425	0.522	0.464	0.315	0.505	0.47	0.545	0.459	0.472	p.Trp78*	78/836	9.3	0.4872	0.0190	0.0553	0.0185
Chr7:80300449	rs3211938	CD36	NM 000072.3	0.0279	0.0819	0.0002	0.033	0.146	0	0	0	0.074	0	0	0	0	0	0	0	0	0.062	0	0	0	0.29	p.Tyr325*	325/473	68.7	0.0495	0.1947	0.2141	0.0120
Chr17:39197499	rs3213755	KRTAP1-1	NM 030967.2	0.1633	0.1844	0.1533	0.195	0.177	0.232	0.264	0.137	0.205	0.188	0.284	0.235	0.192	0.108	0.14	0.071	0.275	0.108	0.341	0.145	0.128	0.233	p.Gln51*	51/178	28.7	0.3114	0.0366	0.0674	0.0160
Chr8:19819724	rs328	LPL	NM 000237.2	0.0863	0.0660	0.0967	0.096	0.053	0.077	0.11	0.123	0.082	0.129	0.093	0.125	0.083	0.113	0.107	0.214	0.112	0.036	0.061	0.091	0.128	0.051	p.Ser474*	474/476	99.6	0.1630	0.0191	0.0274	0.0042
Chr13:53617309	rs34067666	OLFM4	NM 006418.4	0.0114	0.0025	0.0159	0.009	0	0	0	0.026	0	0.006	0	0	0	0.043	0.034	0	0	0	0	0	0.026	0	p.Arg214*	214/511	41.9	0.0165	0.0264	0.0272	0.0004
Chr2:170387886	rs34291832	FASTKD1	NM 024622.3	0.0116	0.0052	0.0149	0.009	0.004	0.008	0	0.018	0.016	0.024	0	0	0.008	0.022	0.028	0	0	0	0	0.018	0.005	0	p.Ser768*	768/848	90.6	0.1529	0.0121	0.0125	0.0002
Chr17:39880966	rs34381648	HAP1	NM 001079870.1	0.0687	0.2009	0.0009	0.056	0.236	0.011	0	0.003	0.131	0	0	0	0.025	0	0.006	0	0	0.309	0.008	0	0.005	0.227	p.Ser599*	599/603	99.3	0.0916	0.1913	0.2237	0.0200
Chr15:69327737	rs34406284	NOX5	NM 001184779.1	0.0282	0.0832	0.0000	0.018	0.081	0	0	0	0.074	0	0	0	0	0	0	0	0	0.062	0	0	0	0.108	p.Trp272*	272/738	36.9	0.0330	0.0697	0.0743	0.0025
Chr6:154567863	rs34427887	OPRM1	NM 001008503.1	0.0529	0.0166	0.0715	0.029	0.006	0.055	0	0.054	0.025	0.065	0	0	0.042	0.016	0.062	0.143	0	0	0.061	0.064	0.061	0	p.Arg401*	401/419	95.7	0.0531	0.0423	0.0488	0.0034
Chr6:31939823	rs34548063	STK19	NM 004197.1	0.0112	0.0329	0.0001	0.010	0.039	0.006	0	0	0.041	0	0	0	0.008	0	0	0	0	0.067	0.008	0	0	0.006	p.Trp17*	17/365	4.7	0.0174	0.0400	0.0414	0.0007
Chr6:150387059	rs34672740	ULBP3	NM 024518.1	0.0183	0.0538	0.0001	0.015	0.063	0.003	0	0	0.049	0	0	0	0	0	0	0	0	0.052	0	0.009	0	0.085	p.Glu110*	110/245	44.9	0.0293	0.0505	0.0532	0.0014
Chr14:102729886	rs34931752	MOK	NM 014226.1	0.0559	0.0890	0.0390	0.043	0.091	0.033	0.021	0.034	0.082	0.041	0.021	0.015	0.042	0.016	0.028	0.107	0.028	0.103	0.008	0.055	0.041	0.085	p.Arg94*	94/420	22.4	0.0778	0.0227	0.0272	0.0023
Chr14:57947421	rs34960436	C14orf105	NM 018168.2	0.0256	0.0068	0.0352	0.016	0.004	0.03	0	0.03	0.008	0.018	0	0	0.033	0.032	0.039	0	0	0.005	0.008	0.055	0.036	0	p.Gln183*	183/297	61.6	0.0330	0.0196	0.0209	0.0007
Chr19:35718891	rs35001809	FAM187B	NM 152481.1	0.0225	0.0093	0.0293	0.015	0.002	0.006	0.002	0.038	0.008	0.047	0	0.005	0	0.038	0.028	0	0	0	0.015	0	0.046	0	p.Trp231*	231/370	62.4	0.0302	0.0236	0.0248	0.0006

Chr22:30891264	rs35032582	*SEC14L4*	NM 001161368.1	0.0321	0.0944	0.0001	0.022	0.091	0.008	0	0.001	0.066	0.006	0	0	0.017	0	0	0	0	0.072	0	0.009	0	0.131	p.Glu134*	134/361	37.1	0.0375	0.0700	0.0757	0.0031
Chr11:102584135	rs35231465	*MMP8*	NM 002424.2	0.0165	0.0045	0.0226	0.011	0.002	0.017	0	0.021	0.008	0.024	0	0	0.008	0.011	0.017	0.071	0	0	0.023	0.018	0.026	0	p.Gln450*	450/468	96.2	0.0211	0.0231	0.0244	0.0007
Chr11:47306630	rs35233100	*MADD*	NM 003682.3	0.0459	0.0118	0.0634	0.022	0.004	0.03	0	0.046	0.016	0.035	0	0	0.008	0.032	0.067	0	0	0	0.038	0.045	0.056	0	p.Arg766*	766/1648	46.5	0.0385	0.0254	0.0275	0.0011
Chr5:94749787	rs35391433	*FAM81B*	NM 152548.2	0.0217	0.0058	0.0293	0.021	0.002	0.033	0	0.042	0.008	0.053	0	0	0.05	0.022	0.039	0	0	0	0.015	0.036	0.061	0	p.Gln144*	144/453	31.8	0.0412	0.0247	0.0267	0.0010
Chr17:4803711	rs35400274	*C17orf107*	NM 001145536.1	0.2054	0.3569	0.1395	0.207	0.445	0.232	0.086	0.132	0.377	0.106	0.082	0.045	0.267	0.134	0.118	0.107	0.135	0.515	0.167	0.273	0.168	0.415	p.Trp152*	152/191	79.6	0.3004	0.1122	0.2049	0.0522
Chr18:21736486	rs35554127	*CABYR*	NM 012189.2	0.0149	0.0440	0.0000	0.012	0.053	0	0	0	0.025	0	0	0	0	0	0	0	0	0.072	0	0	0	0.051	p.Arg341*	341/494	69.0	0.0238	0.0469	0.0488	0.0010
Chr19:2936535	rs35699176	*ZNF77*	NM 021217.2	0.0358	0.0204	0.0437	0.025	0.016	0.036	0	0.045	0.008	0.047	0	0	0.042	0.011	0.073	0.036	0	0.01	0.038	0.027	0.051	0.028	p.Gln100*	100/546	18.3	0.0504	0.0182	0.0202	0.0010
Chr9:136029645	rs35898523	*GBGT1*	NM 021996.4	0.0637	0.0452	0.0731	0.049	0.047	0.044	0.003	0.088	0.041	0.124	0	0.01	0.05	0.075	0.067	0	0	0.052	0.061	0.018	0.102	0.045	p.Tyr121*	121/348	34.8	0.0897	0.0312	0.0370	0.0030
Chr19:19039030	rs36078704	*DDX49*	NM 019070.4	0.0159	0.0468	0.0001	0.010	0.037	0.008	0	0	0.025	0	0	0	0.008	0	0	0	0	0.031	0.008	0.009	0	0.051	p.Arg453*	453/484	93.6	0.0192	0.0241	0.0250	0.0005
Chr16:48130781	rs36102575	*ABCC12*	NM 033226.2	0.0229	0.0098	0.0297	0.018	0.006	0.033	0.002	0.032	0.016	0.035	0.005	0	0.025	0.011	0.039	0	0	0.005	0.053	0.018	0.046	0	p.Trp1024*	1024/1360	75.3	0.0366	0.0180	0.0193	0.0007
Chr3:113955187	rs3732781	*ZNF80*	NM 007136.3	0.2064	0.0635	0.2795	0.187	0.041	0.204	0.168	0.288	0.098	0.282	0.17	0.175	0.142	0.285	0.309	0.214	0.157	0.01	0.258	0.209	0.286	0.034	p.Tyr245*	245/274	89.4	0.2912	0.0540	0.0978	0.0232
Chr15:31294714	rs3784589	*TRPM1*	NM 002420.4	0.0636	0.0793	0.0565	0.050	0.071	0.069	0.023	0.049	0.09	0.041	0.01	0.01	0.117	0.075	0.056	0.107	0.051	0.093	0.03	0.064	0.015	0.034	p.Glu1375*	1375/1604	85.7	0.0989	0.0220	0.0272	0.0026
Chr15:97327393	rs3812907	*SPATA8*	NM 173499.3	0.0835	0.0248	0.1135	0.103	0.016	0.18	0.117	0.112	0.057	0.1	0.124	0.08	0.108	0.118	0.101	0.143	0.152	0	0.258	0.164	0.122	0.006	p.Arg34*	34/106	32.1	0.1822	0.0401	0.0585	0.0096
Chr6:53133964	rs41273878	*ELOVL5*	NM 001242830.1	0.0220	0.0077	0.0293	0.013	0.004	0.017	0	0.028	0.016	0.024	0	0	0.008	0.038	0.034	0	0	0	0	0.045	0.02	0	p.Gly246*	246/263	93.5	0.0247	0.0190	0.0200	0.0005
Chr13:100518634	rs41281112	*CLYBL*	NM 206808.2	0.0205	0.0054	0.0283	0.024	0	0.014	0.042	0.032	0	0.041	0.052	0.04	0	0.048	0.028	0	0.034	0	0.038	0	0.015	0	p.Arg259*	259/341	76.0	0.0449	0.0194	0.0211	0.0008
Chr20:36869005	rs41282820	*KIAA1755*	NM 001029864.1	0.0135	0.0034	0.0187	0.006	0.004	0.011	0	0.009	0.016	0.006	0	0	0.033	0.022	0.011	0	0	0	0	0	0	0	p.Arg510*	510/1201	42.5	0.0119	0.0165	0.0169	0.0002
Chr10:96447562	rs41291550	*CYP2C18*	NM 000772.2	0.0268	0.0735	0.0028	0.029	0.065	0.003	0.051	0.003	0.049	0.012	0.046	0.045	0.008	0	0	0	0.062	0.077	0	0	0	0.062	p.Tyr68*	68/491	13.8	0.0549	0.0315	0.0348	0.0017
Chr9:12709125	rs41302073	*TYRP1*	NM 000550.2	0.0140	0.0409	0.0002	0.006	0.028	0	0	0	0.016	0	0	0	0	0	0	0	0	0	0	0	0	0.068	p.Tyr519*	519/538	96.5	0.0110	0.0524	0.0536	0.0006
ChrX:69478432	rs41310667	*P2RY4*	NM 002565.3	0.0141	0.0063	0.0186	0.010	0.008	0.026	0	0.012	0.02	0.016	0	0	0.033	0.007	0	0.048	0	0.007	0.03	0.012	0.021	0	p.Trp348*	348/366	95.1	X chromosome	0.0286	0.0293	0.0004
Chr14:52735063	rs41407349	*PTGDR*	NM 000953.2	0.0103	0.0304	0.0000	0.006	0.026	0	0	0	0.041	0	0	0	0	0	0	0	0	0.01	0	0	0	0.034	p.Tyr177*	177/360	49.2	0.0101	0.0262	0.0322	0.0031
Chr21:44323720	rs4148974	*NDUFV3*	NM 021075.3	0.0404	0.0093	0.0564	0.060	0.004	0.072	0.082	0.075	0.008	0.065	0.072	0.06	0.075	0.108	0.079	0	0.118	0.005	0.061	0.082	0.061	0	p.Arg200*	200/474	42.2	0.1154	0.1051	0.1187	0.0076
Chr8:39872935	rs4503083	*IDO2*	NM 194294.2	0.1750	0.1097	0.2075	0.247	0.093	0.293	0.399	0.211	0.098	0.282	0.412	0.375	0.325	0.156	0.157	0.214	0.41	0.057	0.295	0.255	0.25	0.131	p.Tyr359*	359/421	85.3	0.3516	0.0655	0.1345	0.0369
Chr12:55641255	rs4522268	*OR6C74*	NM 001005490.1	0.2720	0.1857	0.3162	0.227	0.154	0.271	0.129	0.326	0.18	0.288	0.196	0.08	0.292	0.328	0.309	0.321	0.112	0.18	0.205	0.327	0.372	0.108	p.Arg62*	62/313	19.8	0.3361	0.0471	0.0963	0.0258
Chr9:125239501	rs45579335	*OR1J1*	NM 001004451.1	0.0122	0.0036	0.0166	0.010	0	0.006	0	0.025	0	0.024	0	0	0.008	0.016	0.039	0	0	0	0.008	0	0.026	0	p.Cys235*	235/323	72.8	0.0192	0.0176	0.0182	0.0003
Chr6:36274148	rs45621032	*PNPLA1*	NM 001145716.1	0.0105	0.0023	0.0148	0.011	0	0.019	0.002	0.022	0	0.053	0.005	0	0.017	0	0.017	0.036	0	0	0.03	0.009	0.02	0	p.Tyr402*	402/447	89.9	0.0229	0.0194	0.0204	0.0005
Chr3:32030998	rs4639011	*ZNF860*	NM 001137674.2	0.0723	0.1958	0.0185	0.078	0.295	0.033	0	0.017	0.197	0.024	0	0	0.025	0.011	0.034	0	0	0.258	0	0.082	0.005	0.403	p.Arg143*	143/633	22.6	0.1117	0.2082	0.2590	0.0321
Chr16:72001136	rs4788587	*PKD1L3*	NM 181536.1	0.2054	0.2211	0.1986	0.252	0.23	0.213	0.39	0.181	0.197	0.147	0.402	0.42	0.225	0.247	0.157	0.179	0.343	0.227	0.197	0.218	0.168	0.256	p.Arg789*	789/1733	45.5	0.3553	0.0384	0.0801	0.0217
ChrX:35821127	rs4829392	*MAGEB16*	NM 001099921.1	0.5450	0.5673	0.5323	0.611	0.599	0.584	0.736	0.54	0.551	0.6	0.733	0.713	0.538	0.477	0.511	0.619	0.766	0.63	0.604	0.61	0.568	0.602	p.Arg272*	272/325	83.7	X chromosome	0.0262	0.0716	0.0233
Chr11:5776484	rs4910844	*OR52N4*	NM 001005175.2	0.2092	0.1541	0.2371	0.215	0.163	0.207	0.187	0.273	0.164	0.224	0.222	0.185	0.192	0.366	0.23	0.286	0.152	0.139	0.227	0.2	0.265	0.188	p.Arg172*	172/322	53.4	0.3379	0.0191	0.0382	0.0097
Chr16:70694000	rs4985556	*IL34*	NM 001172771.1	0.0864	0.0239	0.1184	0.056	0.004	0.039	0.045	0.106	0.016	0.094	0.046	0.045	0.042	0.108	0.146	0.143	0.045	0	0.038	0.036	0.071	0	p.Tyr212*	212/242	87.6	0.1044	0.0370	0.0460	0.0046
Chr12:1022569	rs4987208	*RAD52*	NM 134424.2	0.0148	0.0054	0.0190	0.018	0.002	0.083	0	0.011	0.008	0.006	0	0	0.092	0.005	0.022	0	0	0	0.121	0.027	0.01	0	p.Tyr415*	415/419	99.0	0.0321	0.0646	0.0697	0.0027
Chr11:59480952	rs499037	*OR10V1*	NM 001005324.1	0.0383	0.0884	0.0126	0.049	0.1	0.102	0.03	0.007	0.074	0	0.015	0.01	0.083	0.011	0.017	0	0.067	0.113	0.129	0.091	0	0.102	p.Gln123*	123/310	39.7	0.0879	0.0436	0.0525	0.0047
Chr16:20792388	rs52817836	*ACSM3*	NM 005622.3	0.0122	0.0357	0.0001	0.013	0.057	0.003	0	0	0.057	0	0	0	0	0	0	0	0	0.077	0	0.009	0	0.034	p.Trp292*	292/587	49.7	0.0247	0.0470	0.0493	0.0012
Chr19:35719020	rs541169	*FAM187B*	NM 152481.1	0.3254	0.3198	0.3283	0.277	0.297	0.365	0.157	0.314	0.352	0.282	0.18	0.18	0.358	0.285	0.365	0.393	0.107	0.278	0.371	0.364	0.311	0.278	p.Trp188*	188/370	50.8	0.3864	0.0328	0.0767	0.0227
Chr17:72588806	rs545652	*C17orf77*	NM 152460.2	0.2656	0.4469	0.1727	0.207	0.52	0.185	0.038	0.142	0.418	0.112	0.005	0.02	0.2	0.108	0.202	0.071	0.096	0.552	0.121	0.245	0.158	0.557	p.Cys207*	207/244	84.8	0.2592	0.1853	0.3191	0.0821
Chr3:98217178	rs55639376	*OR5K2*	NM 001004737.1	0.1674	0.1353	0.1839	0.127	0.134	0.171	0.068	0.146	0.156	0.182	0.072	0.055	0.2	0.156	0.152	0.179	0.079	0.149	0.136	0.182	0.097	0.102	p.Tyr218*	218/317	68.8	0.2234	0.0170	0.0272	0.0052
Chr9:136131576	rs55727303	*ABO*	NM 020469.2	0.0145	0.0043	0.0196	0.021	0	0.097	0	0.013	0	0.024	0	0	0.117	0.005	0.006	0	0	0	0.121	0.045	0.02	0	p.Trp181*	181/355	51.0	0.0394	0.0704	0.0768	0.0035
Chr11:5068662	rs57026471	*OR52J3*	NM 001001916.2	0.0827	0.0602	0.0942	0.114	0.055	0.193	0.103	0.123	0.09	0.147	0.098	0.1	0.183	0.054	0.124	0.107	0.112	0.031	0.174	0.227	0.168	0.057	p.Arg303*	303/312	97.1	0.2005	0.0271	0.0410	0.0072
Chr1:223285200	rs5744168	*TLR5*	NM 003268.5	0.0443	0.0241	0.0547	0.040	0.035	0.036	0.026	0.055	0.049	0.065	0.036	0.025	0.033	0.065	0.051	0.071	0.017	0.026	0.038	0.036	0.041	0.034	p.Arg392*	392/859	45.6	0.0742	0.0061	0.0071	0.0005
Chr15:55722882	rs57809907	*DYX1C1*	NM 130810.3	0.1999	0.4163	0.0892	0.158	0.494	0.072	0.01	0.091	0.402	0.118	0.01	0.015	0.075	0.086	0.067	0.107	0.006	0.459	0.045	0.1	0.092	0.597	p.Glu417*	417/421	99.0	0.2088	0.2460	0.3676	0.0806
Chr16:81183325	rs59980974	*PKD1L2*	NM 052892.3	0.0732	0.0740	0.0729	0.142	0.114	0.169	0.227	0.083	0.123	0.106	0.206	0.225	0.125	0.086	0.079	0.071	0.253	0.149	0.242	0.127	0.066	0.068	p.Lys1575*	1575/2460	64.0	0.2436	0.0353	0.0562	0.0109
Chr19:49206674	rs601338	*FUT2*	NM 000511.5	0.4935	0.4961	0.4921	0.324	0.49	0.384	0.005	0.429	0.541	0.494	0.015	0	0.375	0.301	0.472	0.286	0	0.433	0.311	0.482	0.474	0.517	p.Trp154*	154/344	44.8	0.3480	0.1609	0.0000	0.1148
Chr20:23805918	rs6049157	*CST2*	NM 001322.2	0.0113	0.0331	0.0001	0.011	0.051	0	0	0	0.041	0	0	0	0	0	0	0	0	0.082	0	0	0	0.023	p.Arg91*	91/142	64.1	0.0229	0.0517	0.0537	0.0011
Chr11:5989223	rs61730422	*OR56A5*	NM 001146033.1	0.0661	0.0195	0.0864	0.038	0.006	0.044	0.009	0.078	0.025	0.065	0.005	0.01	0.058	0.086	0.096	0	0.011	0	0.023	0.055	0.077	0	p.Arg168*	168/314	53.5	0.0650	0.0322	0.0368	0.0024
Chr4:53611484	rs61731313	*ERVMER34-*	NM 001242690.1	0.0182	0.0564	0.0016	0.010	0.041	0.006	0	0	0.008	0	0	0	0.008	0	0	0	0	0.046	0	0.009	0	0.057	p.Trp68*	68/564	12.1	0.0201	0.0346	0.0358	0.0006
Chr19:55019261	rs61737751	*LAIR2*	NM 002288.4	0.0332	0.0104	0.0449	0.029	0	0.069	0.005	0.047	0	0.053	0.005	0	0.067	0.054	0.045	0.036	0.011	0	0.061	0.082	0.041	0	p.Arg76*	76/153	49.7	0.0568	0.0249	0.0281	0.0017
Chr2:18766156	rs61742596	*NT5C1B*	NM 001002006.2	0.0323	0.0067	0.0453	0.051	0.008	0.083	0.066	0.051	0.016	0.035	0.062	0.05	0.067	0.081	0.045	0.071	0.09	0.01	0.106	0.073	0.041	0	p.Ser176*	176/611	28.8	0.0888	0.0176	0.0215	0.0020
Chr1:225140459	rs61745064	*DNAH14*	NM 001145154.1	0.0106	0.0344	0.0000	0.006	0.026	0	0	0.001	0.033	0	0	0	0	0	0.006	0	0	0.015	0	0	0	0.034	p.Glu19*	19/454	4.2	0.0128	0.0224	0.0229	0.0003
Chr6:150212034	rs61746769	*RAET1E*	NM 139165.2	0.0209	0.0617	0.0000	0.013	0.057	0.003	0	0	0.025	0	0	0	0.008	0	0	0	0	0.041	0	0	0	0.097	p.Arg2*	2/264	0.8	0.0229	0.0570	0.0597	0.0014
Chr15:42162467	rs61750839	*SPTBN5*	NM 016642.2	0.0247	0.0051	0.0341	0.017	0	0.017	0	0.042	0	0.029	0	0	0.033	0.081	0.039	0	0	0	0.008	0.009	0.026	0	p.Arg1848*	1848/3640	50.8	0.0330	0.0326	0.0346	0.0011
Chr19:9236969	rs61751875	*OR7G3*	NM 001001958.1	0.0311	0.0876	0.0022	0.034	0.104	0.006	0.037	0.001	0.082	0.006	0.026	0.025	0	0	0	0	0.062	0.108	0.015	0	0	0.114	p.Arg220*	220/313	70.3	0.0614	0.0544	0.0610	0.0035
Chr16:1825982	rs61753375	*EME2*	NM 001010865.1	0.0110	0.0037	0.0147	0.006	0	0.003	0	0.017	0	0.018	0	0	0.008	0.011	0.034	0.036	0	0	0	0	0.005	0	p.Gln387*	387/445	87.0	0.0128	0.0186	0.0192	0.0003
Chr1:152285861	rs61816761	*FLG*	NM 002016.1	0.0141	0.0061	0.0183	0.005	0	0.011	0	0.011	0	0.012	0	0	0.008	0.011	0.017	0	0	0	0.015	0.009	0.005	0	p.Arg501*	501/4062	12.3	0.0110	0.0068	0.0070	0.0001
Chr11:55861650	rs61887097	*OR8I2*	NM 001003750.1	0.0605	0.0218	0.0804	0.039	0.008	0.039	0	0.09	0.008	0.118	0	0	0.042	0.108	0.062	0.071	0	0.005	0.045	0.027	0.077	0.011	p.Tyr289*	289/311	92.9	0.0733	0.0389	0.0453	0.0033
Chr12:113403675	rs61942233	*OAS3*	NM 006187.2	0.0129	0.0045	0.0168	0.006	0.004	0.011	0	0.011	0.008	0.006	0	0	0.008	0.011	0.006	0	0	0.005	0.023	0	0.02	0	p.Arg844*	844/1088	77.6	0.0128	0.0085	0.0087	0.0001
Chr13:21750538	rs61950356	*SKA3*	NM 001166017.1	0.0709	0.0307	0.0912	0.125	0.035	0.138	0.203	0.119	0.074	0.082	0.227	0.205	0.117	0.226	0.073	0.071	0.174	0.036	0.197	0.091	0.097	0.006	p.Arg27*	27/389	6.9	0.2500	0.0463	0.0696	0.0122
Chr2:98779439	rs62154921	*VWA3B*	NM 144992.4	0.0318	0.0105	0.0420	0.020	0.004	0.014	0.007	0.042	0.016	0.076	0.005	0.005	0.025	0.027	0.028	0.107	0.011	0	0.008	0.009	0.031	0	p.Glu372*	372/1295	28.7	0.0394	0.0362	0.0398	0.0018
Chr22:32643460	rs62239058	*SLC5A4*	NM 014227.2	0.0108	0.0027	0.0149	0.007	0	0.014	0	0.015	0	0.018	0	0	0.025	0.005	0.006	0	0	0	0	0.018	0.031	0	p.Glu139*	139/660	21.1	0.0147	0.0149	0.0153	0.0002
Chr1:109650558	rs62623709	*C1orf194*	NM 001122961.1	0.0120	0.0395	0.0000	0.010	0.043	0.003	0	0	0.041	0	0	0	0	0	0	0	0	0.021	0	0.009	0	0.068	p.Tyr49*	49/158	31.0	0.0165	0.0397	0.0412	0.0008
Chr1:47080679	rs6671527	*MOB3C*	NM 145279.4	0.4988	0.5579	0.4685	0.616	0.549	0.53	0.928	0.464	0.549	0.447	0.943	0.945	0.575	0.5	0.416	0.393	0.893	0.557	0.523	0.491	0.5	0.54	p.Arg24*	24/269	8.9	0.4112	0.1384	0.3361	0.1148
Chr7:64452738	rs67047829	*ERV3-1*	NM 001007253.3	0.0670	0.0278	0.0858	0.107	0.026	0.11	0.199	0.087	0.025	0.082	0.149	0.21	0.117	0.091	0.118	0.107	0.242	0.036	0.106	0.109	0.056	0.017	p.Arg223*	223/605	36.9	0.1731	0.0412	0.0592	0.0094
Chr6:117150008	rs6907580	*GPRC6A*	NM 148963.2	0.0698	0.0790	0.0651	0.068	0.077	0.127	0.01	0.077	0.115	0.035	0.015	0	0.133	0.097	0.107	0.071	0.017	0.026	0.159	0.082	0.066	0.108	p.Arg57*	57/927	6.1	0.1264	0.0327	0.0427	0.0052
Chr11:48266736	rs7120775	*OR4X2*	NM 001004727.1	0.1785	0.2687	0.1323	0.167	0.258	0.127	0.163	0.129	0.221	0.118	0.139	0.18	0.192	0.167	0.152	0.107	0.169	0.211	0.076	0.118	0.087	0.335	p.Tyr27*	27/304	8.9	0.2527	0.0304	0.0522	0.0112
Chr19:41381683	rs71358943	*CYP2A7*	NM 000764.2	0.0585	0.0714	0.0520	0.057	0.096	0.083	0.003	0.059	0.074	0.088	0.005	0.005	0.092	0.027	0.039	0.071	0	0.108	0.068	0.091	0.082	0.097	p.Ser467*	467/495	94.3	0.1081	0.0232	0.0289	0.0030
Chr17:39305563	rs71371463	*KRTAP4-5*	NM 033188.3	0.0261	0.0766	0.0002	0.021	0.089	0.006	0	0	0.082	0	0	0	0	0	0	0	0	0.046	0.008	0.009	0	0.142	p.Arg153*	153/182	84.1	0.0421	0.0829	0.0892	0.0035
Chr17:45425287	rs71377306	*C17orf57*	NM 152347.4	0.0398	0.0134	0.0533	0.088	0	0.03	0.199	0.09	0	0.053	0.186	0.265	0.042	0.151	0.045	0.036	0.14	0	0.03	0.018	0.112	0	p.Arg211*	211/974	21.7	0.1493	0.0863	0.1120	0.0141
Chr17:3195603	rs7218125	*OR3A1*	NM 002550.2	0.0216	0.0631	0.0003	0.017	0.063	0.014	0	0.001	0.057	0	0	0	0.025	0	0	0	0	0.057	0.008	0.009	0.005	0.074	p.Lys92*	92/316	29.1	0.0321	0.0382	0.0407	0.0013
Chr6:31125257	rs72856718	*CCHCR1*	NM 001105563.1	0.0748	0.0540	0.0854	0.090	0.063	0.149	0.04	0.116	0.033	0.071	0.062	0.03	0.158	0.183	0.062	0.143	0.028	0.062	0.174	0.109	0.138	0.085	p.Glu41*	41/836	4.9	0.1667	0.0321	0.0450	0.0067
Chr18:658001	rs73366471	*C18orf56*	NM 001012716.2	0.0128	0.0376	0.0002	0.019	0.083	0.003	0	0	0.057	0	0	0	0	0	0	0	0	0.134	0	0.009	0	0.045	p.Gln83*	83/124	66.9	0.0366	0.0790	0.0842	0.0028
Chr7:150027578	rs73474332	*C7orf29*	NM 138434.2	0.0129	0.0379	0.0001	0.012	0.045	0.011	0	0	0.033	0	0	0	0	0	0	0	0	0.036	0.008	0.027	0	0.062	p.Gln29*	29/237	12.2	0.0238	0.0311	0.0326	0.0007
Chr19:373531	rs73489977	*THEG*	NM 016585.3	0.0398	0.1167	0.0003	0.028	0.114	0.014	0	0	0.082	0	0	0	0.025	0	0	0	0	0.103	0	0.018	0	0.148	p.Trp168*	168/380	44.2	0.0504	0.0821	0.0903	0.0045
Chr8:43171055	rs73575237	*POTEA*	NM 001002920.1	0.0254	0.0773	0.0001	0.021	0.089	0.003	0	0.001	0.066	0	0	0	0.008	0	0	0.036	0	0.082	0	0	0	0.114	p.Ser263*	263/453	58.1	0.0366	0.0625	0.0677	0.0028
Chr11:2321829	rs74048215	*C11orf21*	NM 001142946.1	0.0151	0.0491	0.0003	0.011	0.041	0.008	0	0	0.041	0	0	0	0.008	0	0	0	0	0.021	0.008	0.009	0	0.062	p.Trp69*	69/179	38.5	0.0192	0.0314	0.0327	0.0007
Chr1:155291263	rs74118444	*RUSC1-AS1*	NM 001039517.1	0.0173	0.0529	0.0013	0.015	0.063	0.003	0	0	0.107	0	0	0	0	0	0	0	0	0.057	0	0.009	0	0.04	p.Ser6*	6/237	2.5	0.0238	0.0623	0.0659	0.0019
Chr3:193052769	rs74437357	*ATP13A5*	NM 198505.2	0.0215	0.0054	0.0298	0.014	0.002	0.008	0	0.036	0.008	0.024	0	0	0.017	0.048	0.067	0	0	0	0	0.009	0.01	0	p.Gln355*	355/1219	29.1	0.0247	0.0307	0.0323	0.0008
Chr5:1240757	rs7447815	*SLC6A18*	NM 182632.2	0.3644	0.3482	0.3728	0.340	0.382	0.373	0.245	0.369	0.369	0.382	0.232	0.16	0.392	0.301	0.438	0.393	0.354	0.448	0.364	0.364	0.357	0.318	p.Tyr319*	319/629	50.7	0.4240	0.0240	0.0622	0.0196
Chr19:55526026	rs74697203	*GP6*	NM 001083899.1	0.0138	0.0417	0.0004	0.010	0.043	0.003	0	0	0.066	0	0	0	0	0	0	0	0	0.031	0	0.009	0	0.04	p.Trp429*	429/621	69.1	0.0201	0.0376	0.0391	0.0008
Chr19:3789321	rs74830030	*MATK*	NM 002378.3	0.0195	0.0574	0.0001	0.013	0.051	0.008	0	0	0.041	0	0	0	0	0	0	0	0	0.062	0.008	0.018	0	0.045	p.Arg9*	9/509	1.8	0.0256	0.0337	0.0354	0.0008
Chr12:7475081	rs7485773	*ACSM4*	NM 001080454.1	0.0597	0.0927	0.0442	0.049	0.087	0.052	0.01	0.053	0.09	0.053	0.005	0	0.05	0.043	0.028	0.107	0.028	0.119	0.068	0.036	0.077	0.051	p.Gln357*	357/581	61.4	0.0989	0.0225	0.0275	0.0026
Chr17:45452257	rs74969489	*C17orf57*	NM 001195192.1	0.0120	0.0023	0.0170	0.006	0.002	0.006	0	0.015	0.008	0.018	0	0	0	0.005	0.022	0	0	0	0	0.018	0.015	0	p.Lys337*	337/785	42.9	0.0128	0.0108	0.0111	0.0001
Chr16:81242198	rs7499011	*PKD1L2*	NM 001076780.1	0.2902	0.1420	0.3640	0.196	0.079	0.301	0.002	0.367	0.115	0.312	0	0.005	0.333	0.317	0.337	0.429	0	0.041	0.242	0.336	0.48	0.097	p.Gln220*	220/992	22.2	0.2555	0.1556	0.2810	0.0743
Chr1:12919891	rs75411676	*PRAMEF2*	NM 023014.1	0.0379	0.0243	0.0449	0.078	0.008	0.028	0.217	0.044	0.008	0.047	0.247	0.205	0.033	0.065	0.034	0.036	0.197	0.005	0.03	0.018	0.031	0.011	p.Glu211*	211/475	44.4	0.1529	0.0971	0.1226	0.0141
Chr11:55371381	rs75423534	*OR4C11*	NM 001004700.2	0.0986	0.0289	0.1365	0.074	0.002	0.113	0.007	0.152	0.008	0.129	0.01	0	0.167	0.247	0.146	0.107	0.011	0	0.076	0.1	0.092	0	p.Gln157*	157/311	50.5	0.0687	0.0768	0.1004	0.0128
Chr2:26700289	rs75624587	*OTOF*	NM 004802.3	0.0346	0.1015	0.0007	0.022	0.087	0.008	0	0.001	0.082	0	0	0	0	0	0	0	0	0.108	0.008	0.018	0.005	0.068	p.Glu54*	54/1231	4.4	0.0412	0.0619	0.0667	0.0026
Chr17:67189302	rs75656517	*ABCA10*	NM 080282.3	0.0157	0.0458	0.0002	0.011	0.043	0.006	0	0	0.049	0	0	0	0.008	0	0	0	0	0.031	0	0.009	0	0.051	p.Arg577*	577/1544	37.4	0.0192	0.0310	0.0323	0.0007
Chr11:59245593	rs75898556	*OR4D10*	NM 001004705.1	0.0473	0.1174	0.0113	0.076	0.175	0.122	0.051	0.011	0.164	0	0.057	0.03	0.075	0.022	0.017	0	0.067	0.175	0.167	0.118	0.005	0.182	p.Glu231*	231/312	74.0	0.1364	0.0647	0.0845	0.0106
Chr14:21500218	rs76101114	*TPPP2*	NM 173846.4	0.0266	0.0756	0.0015	0.030	0.13	0.003	0	0.001	0.107	0	0	0	0	0	0	0	0	0.139	0	0.009	0.005	0.136	p.Tyr165*	165/171	96.5	0.0476	0.0992	0.1096	0.0057
Chr11:55406022	rs76160133	*OR4P4*	NM 001004124.1	0.1342	0.1089	0.1479	0.188	0.073	0.21	0.369	0.116	0.057	0.141	0.33	0.385	0.208	0.048	0.112	0.143	0.393	0.041	0.212	0.209	0.158	0.119	p.Tyr63*	63/313	20.1	0.1584	0.0841	0.1474	0.0346
Chr6:150343126	rs76294770	*RAET1L*	NM 130900.2	0.0164	0.0481	0.0001	0.012	0.055	0	0	0	0.041	0	0	0	0	0	0	0	0	0.046	0	0	0	0.074	p.Tyr113*	113/247	45.7	0.0247	0.0466	0.0487	0.0011
Chr1:198505831	rs76330087	*ATP6V1G3*	NM 133326.1	0.0197	0.0576	0.0002	0.017	0.071	0.006	0	0	0.016	0	0	0	0.017	0	0	0	0	0.077	0	0	0	0.102	p.Arg36*	36/60	60.0	0.0302	0.0657	0.0695	0.0020
Chr3:186461524	rs76438938	*KNG1*	NM 000893.3	0.0204	0.0066	0.0274	0.015	0.004	0.008	0.005	0.032	0.016	0.024	0.005	0.01	0	0	0.043	0.034	0	0	0	0.027	0.031	0	p.Arg412*	412/428	96.3	0.0275	0.0163	0.0172	0.0004
Chr4:155675	rs7692722	*ZNF718*	NM 001039127.3	0.0146	0.0445	0.0001	0.014	0.059	0.003	0	0	0.049	0	0	0	0	0	0	0	0	0.067	0	0.009	0	0.057	p.Cys337*	337/416	81.0	0.0275	0.0437	0.0459	0.0012
Chr11:62850775	rs77002186	*SLC22A24*	NM 001136506.2	0.0158	0.0043	0.0207	0.039	0	0.028	0.105	0.021	0	0.035	0.093	0.095	0.017	0.011	0.011	0.036	0.129	0	0.045	0.018	0.026	0	p.Gln409*	409/553	74.0	0.0769	0.0431	0.0494	0.0033
Chr7:99817585	rs7786505	*PVRIG*	NM 024070.3	0.2227	0.2086	0.2299	0.158	0.201	0.102	0.058	0.234	0.205	0.241	0.046	0.05	0.142	0.194	0.253	0.179	0.079	0.155	0.068	0.1	0.255	0.25	p.Glu18*	18/327	5.5	0.2491	0.0428	0.0721	0.0153
Chr7:34917740	rs7809642	*NPSR1*	NM 207173.1	0.2046	0.1253	0.2452	0.168	0.093	0.144	0.152	0.24	0.115	0.282	0.119	0.145	0.142	0.204	0.315	0.25	0.197	0.108	0.129	0.164	0.168	0.062	p.Arg360*	360/378	95.2	0.2573	0.0332	0.0584	0.0130
Chr15:22368957	rs78256866	*OR4M2*	NM 001004719.2	0.0185	0.0204	0.0174	0.065	0.033	0.028	0.166	0.028	0.025	0.029	0.201	0.115	0.025	0.038	0.017	0	0.185	0.036	0.015	0.045	0.031	0.034	p.Arg128*	128/314	40.8	0.1190	0.0695	0.0849	0.0083
Chr14:94935628	rs78283108	*SERPINA9*	NM 001042518.1	0.0188	0.0572	0.0012	0.015	0.053	0.017	0	0.001	0.057	0	0	0	0.008	0	0	0	0	0.062	0.023	0.018	0.005	0.04	p.Gln102*	102/336	30.4	0.0302	0.0304	0.0322	0.0009
Chr11:18159669	rs78408237	*MRGPRX3*	NM 054031.3	0.0542	0.0125	0.0756	0.040	0.01	0.069	0.009	0.07	0.025	0.047	0.005	0.015	0.05	0.048	0.056	0.107	0.006	0.01	0.045	0.118	0.117	0	p.Trp307*	307/323	95.0	0.0714	0.0359	0.0426	0.0035
Chr12:31291163	rs78835222	*OVOS2*	XM 002343151.2	0.1377	0.2128	0.1040	0.180	0.234	0.135	0.271	0.098	0.205	0.124	0.304	0.215	0.142	0.086	0.101	0.036	0.298	0.211	0.189	0.064	0.092	0.278	p.Arg667*	667/1395	47.8	0.2903	0.0520	0.0894	0.0197
Chr16:89645368	rs78990556	*CPNE7*	NM 014427.4	0.0150	0.0439	0.0002	0.016	0.067	0.006	0	0	0.008	0	0	0	0.017	0	0	0	0	0.088	0	0	0	0.085	p.Ser154*	154/634	24.3	0.0284	0.0641	0.0675	0.0018
Chr10:102056016	rs7904983	*PKD2L1*	NM 016112.2	0.0573	0.1668	0.0012	0.057	0.23	0.03	0	0.001	0.205	0	0	0	0.033	0	0	0	0	0.273	0.023	0.036	0.005	0.199	p.Arg407*	407/806	50.5	0.0925	0.1590	0.1896	0.0182
Chr11:4598956	rs79448530	*C11orf40*	NM 144663.1	0.0439	0.0123	0.0600	0.029	0.004	0.064	0	0.051	0.016	0.065	0	0	0.1	0.022	0.028	0.036	0	0	0.053	0.036	0.092	0	p.Trp32*	32/218	14.7	0.0549	0.0352	0.0396	0.0023
Chr13:46288017	rs79707842	*SPERT*	NM 152719.1	0.0394	0.0092	0.0549	0.074	0.012	0.064	0.119	0.084	0.016	0.071	0.139	0.1	0.075	0.091	0.084	0	0.118	0.021	0.083	0.027	0.102	0	p.Ser286*	286/449	63.7	0.1401	0.0307	0.0391	0.0043
Chr1:247654493	rs79802002	*OR2W5*	NM 001004698.2	0.0141	0.0404	0.0006	0.011	0.041	0.011	0	0	0.025	0	0	0	0	0	0	0	0	0.031	0.015	0.018	0	0.062	p.Gly22*	22/321	6.9	0.0201	0.0293	0.0306	0.0006
Chr3:113955726	rs79892855	*ZNF80*	NM 007136.3	0.0145	0.0427	0.0000	0.013	0.053	0.006	0	0	0.025	0	0	0	0	0	0	0	0	0.067	0.008	0.009	0	0.057	p.Arg66*	66/274	24.1	0.0256	0.0399	0.0418	0.0010
Chr3:46786162	rs79958632	*PRSS45*	NM 199183.2	0.0258	0.0779	0.0002	0.023	0.089	0.006	0	0.005	0.09	0.012	0	0	0.008	0.005	0.006	0	0	0.057	0	0.009	0	0.125	p.Trp28*	28/229	12.2	0.0458	0.0668	0.0724	0.0030
Chr13:46287373	rs80072371	*SPERT*	NM 152719.1	0.0749	0.1272	0.0482	0.056	0.136	0.05	0	0.049	0.107	0.029	0	0	0.058	0.07	0.056	0.071	0	0.134	0.045	0.045	0.036	0.159	p.Cys71*	71/449	15.8	0.1026	0.0398	0.0491	0.0049
Chr17:33772658	rs8072510	*SLFN13*	NM 144682.5	0.1001	0.0994	0.1005	0.109	0.12	0.052	0.163	0.09	0.148	0.059	0.186	0.12	0.05	0.102	0.079	0.143	0.185	0.067	0.03	0.082	0.107	0.159	p.Tyr14*	14/898	1.6	0.1841	0.0241	0.0353	0.0057
Chr19:52130931	rs8108078	*SIGLEC5*	NM 003830.2	0.0458	0.1336	0.0009	0.038	0.15	0.019	0	0.001	0.098	0	0	0	0.025	0	0	0	0	0.124	0.008	0.027	0.005	0.216	p.Arg356*	356/552	64.5	0.0659	0.1134	0.1282	0.0084
Chr6:132938842	rs8192646	*TAAR2*	NM 001033080.1	0.0192	0.0141	0.0219	0.050	0.018	0.006	0.161	0.009	0.016	0.006	0.149	0.22	0.008		0.006	0.036	0.107	0.031		0.009	0.02	0.006	p.Trp168*	168/352	47.7	0.0861	0.0994	0.1156	0.0090
Chr1:48708228	rs850763	*SLC5A9*	NM 001011547.2	0.1481	0.1337	0.1555	0.105	0.134	0.113	0.005	0.158	0.107	0.147	0	0	0.092	0.161	0.152	0.143	0.017	0.149	0.121	0.127	0.173	0.136	p.Glu593*	593/682	87.0	0.1777	0.0344	0.0502	0.0082
Chr1:158549492	rs863362	*OR10X1*	NM 001004477.1	0.4863	0.5672	0.4449	0.535	0.593	0.417	0.621	0.488	0.607	0.524	0.649	0.62	0.417	0.591	0.433	0.464	0.59	0.577	0.348	0.5	0.413	0.602	p.Trp66*	66/327	20.2	0.4615	0.0334	0.0957	0.0322
Chr21:31744127	rs877346	*KRTAP13-2*	NM 181621.3	0.3940	0.2152	0.4856	0.310	0.161	0.351	0.229	0.447	0.18	0.5	0.237	0.225	0.325	0.452	0.506	0.357	0.225	0.129	0.326	0.409	0.357	0.182	p.Cys135*	135/176	76.7	0.3791	0.0645	0.1517	0.0467
Chr1:161476204	rs9427397	*FCGR2A*	NM 001136219.1	0.0968	0.0291	0.1315	0.055	0.016	0.075	0.002	0.111	0.016	0.147	0.005	0	0.108	0.113	0.051	0.107	0	0.026	0.053	0.064	0.133	0.006	p.Gln63*	63/318	19.8	0.0989	0.0469	0.0581	0.0058
Chr9:139634495	rs9886752	*LCN10*	NM 001001712.2	0.1626	0.1806	0.1533	0.201	0.201	0.213	0.264	0.146	0.139	0.135	0.294	0.3	0.275	0.108	0.18	0.214	0.191	0.299	0.205	0.155	0.153	0.136	p.Gln161*	161/201	80.1	0.3132	0.0267	0.0508	0.0124
Chr19:57642782	rs9973206	*USP29*	NM 020903.2	0.8830	0.9746	0.8360	0.933	0.994	0.925	1	0.847	0.975	0.871	1	1	0.908	0.833	0.882	0.75	1	1	0.977	0.882	0.821	1	p.Tyr913*	913/923	98.9	0.1062	0.0887	0.1157	0.0148
Chr11:104763117	rs497116	*CASP12*	NP 001177945.2	No data	No data	No data	0.960	0.837	0.983	0.998	1	0.885	1	0.995	1	0.975	1	1	1	1	0.789	1	0.973	1	0.858	p.Arg125*	125/341	36.7	0.0705	0.1180	0.1340	0.0091
Chr11:66328095	rs1815739	*ACTN3*	NM 001104.3.	0.3591	0.1789	0.4505	0.375	0.112	0.528	0.428	0.431	0.197	0.476	0.412	0.385	0.5	0.349	0.489	0.393	0.494	0.082	0.583	0.491	0.423	0.085	p.Arg577*	577/901	64.0	0.4505	0.0953	0.2376	0.0787

Table S1

Summary statistics and allele information of 246 PTC mutations

dbSNP ID	Gene name	SIFT results	Mutationtaster results	NMD
rs10009430	C4orf33	DAMAGING	DISEASE CAUSING	NMD
rs10181739	GNLY	DAMAGING	POSSIBLY PLYMORPHISM	NMD
rs10237332	ZNF727	DAMAGING	DISEASE CAUSING	NMD
rs1023840	HEATR7B2	DAMAGING	POSSIBLY PLYMORPHISM	NMD
rs10261977	SSPO	DAMAGING	NOT FOUND	truncated protein (might cause NMD)
rs10409173	DPRX	DAMAGING	PLYMORPHISM	truncated protein (might cause NMD)
rs10423255	DHDH	DAMAGING	POSSIBLY PLYMORPHISM	NMD
rs1043149	ZACN	DAMAGING	POSSIBLY PLYMORPHISM	NMD
rs1043261	IL17RB	DAMAGING	POSSIBLY PLYMORPHISM	truncated protein (might cause NMD)
rs1044261	IDI2	DAMAGING	POSSIBLY PLYMORPHISM	truncated protein (might cause NMD)
rs10471773	CCDC125	DAMAGING	DISEASE CAUSING	NMD
rs10491178	ABCA10	DAMAGING	POSSIBLY PLYMORPHISM	NMD
rs10838851	OR4X1	DAMAGING	POSSIBLY PLYMORPHISM	truncated protein (might cause NMD)
rs10888281	OR2L8	DAMAGING	POSSIBLY PLYMORPHISM	truncated protein (might cause NMD)
rs10981589	ZNF883	DAMAGING	NOT FOUND	truncated protein (might cause NMD)
rs11071990	CALML4	DAMAGING	POSSIBLY PLYMORPHISM	NMD
rs11089781	APOL3	DAMAGING	POSSIBLY PLYMORPHISM	NMD
rs111350153	ZIM3	DAMAGING	DISEASE CAUSING	truncated protein (might cause NMD)
rs111696697	UGT2A1	DAMAGING	POSSIBLY PLYMORPHISM	NMD
rs112033303	COQ2	DAMAGING	DISEASE CAUSING	NMD
rs112050262	SULT1C3	DAMAGING	POSSIBLY PLYMORPHISM	NMD
rs11228710	OR5AR1	DAMAGING	POSSIBLY PLYMORPHISM	truncated protein (might cause NMD)
rs11231341	SLC22A24	DAMAGING	POSSIBLY PLYMORPHISM	NMD
rs1130385	HLA-DQB1	DAMAGING	DISEASE CAUSING	NMD
rs113710768	TAS2R43	DAMAGING	PLYMORPHISM	truncated protein (might cause NMD)
rs114232158	HEATR4	DAMAGING	DISEASE CAUSING	NMD
rs114358105	UMODL1	DAMAGING	POSSIBLY PLYMORPHISM	NMD
rs114429815	GJB4	DAMAGING	DISEASE CAUSING	truncated protein (might cause NMD)
rs114730569	ABHD14B	DAMAGING	DISEASE CAUSING	NMD
rs114882604	C10orf90	DAMAGING	DISEASE CAUSING	NMD
rs11542462	SDR42E1	DAMAGING	POSSIBLY PLYMORPHISM	truncated protein (might cause NMD)
rs115687886	METTL7B	DAMAGING	POSSIBLY PLYMORPHISM	truncated protein (might cause NMD)
rs115917139	TTLL3	DAMAGING	NOT FOUND	truncated protein (might cause NMD)
rs116268446	TIAM2	DAMAGING	POSSIBLY PLYMORPHISM	truncated protein (might cause NMD)
rs116389032	PSRC1	NOT FOUND	DISEASE CAUSING	NMD
rs116939015	ANKRD30A	DAMAGING	DISEASE CAUSING	NMD
rs117366703	OR8K3	DAMAGING	DISEASE CAUSING	truncated protein (might cause NMD)
rs117752382	PXDNL	DAMAGING	DISEASE CAUSING	NMD
rs118004742	C17orf57	DAMAGING	POSSIBLY PLYMORPHISM	NMD
rs11913840	PRODH	DAMAGING	POSSIBLY PLYMORPHISM	NMD
rs12077871	COL9A2	DAMAGING	POSSIBLY PLYMORPHISM	NMD
rs12139100	PLA2G2C	DAMAGING	POSSIBLY PLYMORPHISM	NMD
rs12240276	AKR1E2	DAMAGING	POSSIBLY PLYMORPHISM	truncated protein (might cause NMD)
rs1227794	ZNF790	DAMAGING	POSSIBLY PLYMORPHISM	truncated protein (might cause NMD)
rs12409540	OR10J1	DAMAGING	POSSIBLY PLYMORPHISM	truncated protein (might cause NMD)
rs12484086	RFPL1	DAMAGING	POSSIBLY PLYMORPHISM	truncated protein (might cause NMD)
rs12520799	C5orf20	DAMAGING	POSSIBLY PLYMORPHISM	truncated protein (might cause NMD)
rs12568784	FLG2	DAMAGING	PLYMORPHISM	truncated protein (might cause NMD)
rs12829245	PRB4	DAMAGING	DISEASE CAUSING	NMD
rs12925771	PKD1L2	DAMAGING	NOT FOUND	truncated protein (might cause NMD)
rs12986075	PSG1	DAMAGING	POSSIBLY PLYMORPHISM	NMD
rs13338754	CES5A	DAMAGING	POSSIBLY PLYMORPHISM	NMD
rs1343879	MAGEE2	DAMAGING	POSSIBLY PLYMORPHISM	truncated protein (might cause NMD)
rs137943557	KRTAP4-8	DAMAGING	DISEASE CAUSING	truncated protein (might cause NMD)
rs138377917	PSCA	DAMAGING	DISEASE CAUSING	truncated protein (might cause NMD)
rs139948519	CCT8L2	DAMAGING	DISEASE CAUSING	truncated protein (might cause NMD)
rs140171913	OR5H1	DAMAGING	DISEASE CAUSING	truncated protein (might cause NMD)
rs140363602	AMY1C	DAMAGING	DISEASE CAUSING	NMD
rs1404453	ZNF117	DAMAGING	POSSIBLY PLYMORPHISM	truncated protein (might cause NMD)
rs140871032	NOTCH2NL	DAMAGING	DISEASE CAUSING	NMD
rs141833592	RHD	DAMAGING	POSSIBLY PLYMORPHISM	NMD
rs143899788	HHLA3	DAMAGING	PLYMORPHISM	truncated protein (might cause NMD)
rs145014075	CYP2A6	DAMAGING	DISEASE CAUSING	truncated protein (might cause NMD)
rs1459101	OR4C16	DAMAGING	POSSIBLY PLYMORPHISM	truncated protein (might cause NMD)
rs146018563	TCP10L2	DAMAGING	DISEASE CAUSING	truncated protein (might cause NMD)
rs146753414	KRT83	DAMAGING	DISEASE CAUSING	NMD
rs147505098	PKD1L3	DAMAGING	NOT FOUND	truncated protein (might cause NMD)
rs1476860	OR1B1	DAMAGING	POSSIBLY PLYMORPHISM	truncated protein (might cause NMD)
rs147765240	LCE4A	DAMAGING	DISEASE CAUSING	truncated protein (might cause NMD)
rs148998855	OR10Z1	DAMAGING	DISEASE CAUSING	truncated protein (might cause NMD)
rs150843673	TMC3	DAMAGING	DISEASE CAUSING	truncated protein (might cause NMD)
rs151171939	MAGEC3	DAMAGING	DISEASE CAUSING	NMD
rs16910526	CLEC7A	DAMAGING	POSSIBLY PLYMORPHISM	truncated protein (might cause NMD)

rs16919417	OR2D2	DAMAGING	DISEASE CAUSING	truncated protein (might cause NMD)
rs16930998	OR51I1	DAMAGING	POSSIBLY PLYMORPHISM	truncated protein (might cause NMD)
rs16982743	SIGLEC12	DAMAGING	POSSIBLY PLYMORPHISM	NMD
rs16987628	RFPL1	DAMAGING	DISEASE CAUSING	truncated protein (might cause NMD)
rs17001893	OR7G3	DAMAGING	DISEASE CAUSING	truncated protein (might cause NMD)
rs17147990	HTN3	DAMAGING	PLYMORPHISM	truncated protein (might cause NMD)
rs17174638	OPRM1	DAMAGING	DISEASE CAUSING	NMD
rs17184009	OR10C1	NOT FOUND	POSSIBLY PLYMORPHISM	truncated protein (might cause NMD)
rs17292725	STARD6	DAMAGING	POSSIBLY PLYMORPHISM	NMD
rs17547284	OR5M11	DAMAGING	POSSIBLY PLYMORPHISM	truncated protein (might cause NMD)
rs17602729	AMPD1	NOT FOUND	POSSIBLY PLYMORPHISM	NMD
rs17610181	NACA2	DAMAGING	POSSIBLY PLYMORPHISM	truncated protein (might cause NMD)
rs17855778	ZNF83	DAMAGING	POSSIBLY PLYMORPHISM	truncated protein (might cause NMD)
rs1790218	SLC22A10	DAMAGING	POSSIBLY PLYMORPHISM	NMD
rs1811890	DHRS4L2	DAMAGING	POSSIBLY PLYMORPHISM	NMD
rs183603441	AGPHD1	DAMAGING	DISEASE CAUSING	NMD
rs1861050	CC2D2A	NOT FOUND	DISEASE CAUSING	truncated protein (might cause NMD)
rs1894360	CSAG1	DAMAGING	POSSIBLY PLYMORPHISM	NMD
rs199825147	SPATA31C1	NOT FOUND	NOT FOUND	truncated protein (might cause NMD)
rs200375190	POTEH	NOT FOUND	POSSIBLY PLYMORPHISM	NMD
rs201685025	CDRT1	NOT FOUND	DISEASE CAUSING	NMD
rs2039381	IFNE	DAMAGING	POSSIBLY PLYMORPHISM	truncated protein (might cause NMD)
rs2176186	C2orf83	DAMAGING	PLYMORPHISM	truncated protein (might cause NMD)
rs2233919	SMUG1	DAMAGING	DISEASE CAUSING	NMD
rs2235197	UNC93A	DAMAGING	POSSIBLY PLYMORPHISM	NMD
rs2270416	CDH15	DAMAGING	PROBABLY POLYMORPHISM	truncated protein (might cause NMD)
rs2272754	ZC3H3	DAMAGING	DISEASE CAUSING	NMD
rs2273865	LGALS8	DAMAGING	POSSIBLY PLYMORPHISM	NMD
rs2285943	DNAH11	DAMAGING	POSSIBLY PLYMORPHISM	NMD
rs2293766	ZAN	DAMAGING	POSSIBLY PLYMORPHISM	NMD
rs2298553	MS4A12	DAMAGING	POSSIBLY PLYMORPHISM	NMD
rs2301384	H2BFM	DAMAGING	POSSIBLY PLYMORPHISM	NMD
rs233303	KRTAP10-6	DAMAGING	POSSIBLY PLYMORPHISM	truncated protein (might cause NMD)
rs237520	UBE2NL	DAMAGING	POSSIBLY PLYMORPHISM	truncated protein (might cause NMD)
rs2647574	OR51Q1	DAMAGING	POSSIBLY PLYMORPHISM	truncated protein (might cause NMD)
rs2708381	TAS2R46	DAMAGING	POSSIBLY PLYMORPHISM	truncated protein (might cause NMD)
rs2736911	ARMS2	DAMAGING	POSSIBLY PLYMORPHISM	NMD
rs2781377	SYNE2	DAMAGING	POSSIBLY PLYMORPHISM	NMD
rs28413581	ODF3L1	DAMAGING	DISEASE CAUSING	NMD
rs28502153	GAB4	DAMAGING	POSSIBLY PLYMORPHISM	NMD
rs28602966	C18orf56	DAMAGING	DISEASE CAUSING	NMD
rs3130453	CCHCR1	NOT FOUND	POSSIBLY PLYMORPHISM	NMD
rs3211938	CD36	DAMAGING	POSSIBLY PLYMORPHISM	NMD
rs3213755	KRTAP1-1	DAMAGING	POSSIBLY PLYMORPHISM	truncated protein (might cause NMD)
rs328	LPL	DAMAGING	POSSIBLY PLYMORPHISM	truncated protein (might cause NMD)
rs34067666	OLFM4	DAMAGING	DISEASE CAUSING	NMD
rs34291832	FASTKD1	DAMAGING	DISEASE CAUSING	NMD
rs34381648	HAP1	DAMAGING	PLYMORPHISM	truncated protein (might cause NMD)
rs34406284	NOX5	DAMAGING	POSSIBLY PLYMORPHISM	NMD
rs34427887	OPRM1,IPCEF1	NOT FOUND	POSSIBLY PLYMORPHISM	truncated protein (might cause NMD)
rs34548063	STK19	NOT FOUND	DISEASE CAUSING	NMD
rs34672740	ULBP3	DAMAGING	DISEASE CAUSING	NMD
rs34931752	MOK	DAMAGING	POSSIBLY PLYMORPHISM	NMD
rs34960436	C14orf105	DAMAGING	POSSIBLY PLYMORPHISM	NMD
rs35001809	FAM187B	DAMAGING	POSSIBLY PLYMORPHISM	truncated protein (might cause NMD)
rs35032582	SEC14L4	DAMAGING	POSSIBLY PLYMORPHISM	NMD
rs35231465	MMP8	DAMAGING	DISEASE CAUSING	truncated protein (might cause NMD)
rs35233100	MADD	NOT FOUND	POSSIBLY PLYMORPHISM	NMD
rs35391433	FAM81B	DAMAGING	POSSIBLY PLYMORPHISM	NMD
rs35400274	C17orf107	DAMAGING	POSSIBLY PLYMORPHISM	truncated protein (might cause NMD)
rs35554127	CABYR	DAMAGING	DISEASE CAUSING	NMD
rs35699176	ZNF77	DAMAGING	POSSIBLY PLYMORPHISM	truncated protein (might cause NMD)
rs35898523	GBGT1	NOT FOUND	POSSIBLY PLYMORPHISM	truncated protein (might cause NMD)
rs36078704	DDX49	DAMAGING	DISEASE CAUSING	truncated protein (might cause NMD)
rs36102575	ABCC12	DAMAGING	POSSIBLY PLYMORPHISM	NMD
rs3732781	ZNF80	DAMAGING	POSSIBLY PLYMORPHISM	truncated protein (might cause NMD)
rs3784589	TRPM1	DAMAGING	POSSIBLY PLYMORPHISM	truncated protein (might cause NMD)
rs3812907	SPATA8	DAMAGING	POSSIBLY PLYMORPHISM	NMD
rs41273878	ELOVL5	NOT FOUND	NOT FOUND	truncated protein (might cause NMD)
rs41281112	CLYBL	DAMAGING	NOT FOUND	truncated protein (might cause NMD)
rs41282820	KIAA1755	DAMAGING	DISEASE CAUSING	NMD
rs41291550	CYP2C18	DAMAGING	POSSIBLY PLYMORPHISM	NMD
rs41302073	TYRP1	DAMAGING	POSSIBLY PLYMORPHISM	truncated protein (might cause NMD)
rs41310667	P2RY4	DAMAGING	DISEASE CAUSING	truncated protein (might cause NMD)

rs41407349	PTGDR	DAMAGING	DISEASE CAUSING	NMD
rs4148974	NDUFV3	DAMAGING	POSSIBLY PLYMORPHISM	NMD
rs4503083	IDO2	DAMAGING	POSSIBLY PLYMORPHISM	truncated protein (might cause NMD)
rs4522268	OR6C74	DAMAGING	POSSIBLY PLYMORPHISM	truncated protein (might cause NMD)
rs45579335	OR1J1	DAMAGING	DISEASE CAUSING	truncated protein (might cause NMD)
rs45621032	PNPLA1	DAMAGING	DISEASE CAUSING	truncated protein (might cause NMD)
rs4639011	ZNF860	DAMAGING	POSSIBLY PLYMORPHISM	truncated protein (might cause NMD)
rs4788587	PKD1L3	NOT FOUND	NOT FOUND	truncated protein (might cause NMD)
rs4829392	MAGEB16	DAMAGING	POSSIBLY PLYMORPHISM	truncated protein (might cause NMD)
rs4910844	OR52N4	DAMAGING	POSSIBLY PLYMORPHISM	truncated protein (might cause NMD)
rs4985556	IL34	DAMAGING	POSSIBLY PLYMORPHISM	truncated protein (might cause NMD)
rs4987208	RAD52	DAMAGING	POSSIBLY PLYMORPHISM	truncated protein (might cause NMD)
rs499037	OR10V1	DAMAGING	POSSIBLY PLYMORPHISM	truncated protein (might cause NMD)
rs52817836	ACSM3	DAMAGING	DISEASE CAUSING	NMD
rs541169	FAM187B	DAMAGING	POSSIBLY PLYMORPHISM	NMD
rs545652	C17orf77	DAMAGING	PLYMORPHISM	truncated protein (might cause NMD)
rs55639376	OR5K2	DAMAGING	POSSIBLY PLYMORPHISM	truncated protein (might cause NMD)
rs55727303	ABO	DAMAGING	NOT FOUND	truncated protein (might cause NMD)
rs57026471	OR52J3	DAMAGING	PLYMORPHISM	truncated protein (might cause NMD)
rs5744168	TLR5	DAMAGING	POSSIBLY PLYMORPHISM	truncated protein (might cause NMD)
rs57809907	DYX1C1	DAMAGING	PLYMORPHISM	truncated protein (might cause NMD)
rs59980974	PKD1L2	DAMAGING	NOT FOUND	truncated protein (might cause NMD)
rs601338	FUT2	DAMAGING	POSSIBLY PLYMORPHISM	truncated protein (might cause NMD)
rs6049157	CST2	DAMAGING	DISEASE CAUSING	NMD
rs61730422	OR56A5	DAMAGING	NOT FOUND	truncated protein (might cause NMD)
rs61731313	ERVMER34-1	DAMAGING	DISEASE CAUSING	truncated protein (might cause NMD)
rs61737751	LAIR2	DAMAGING	POSSIBLY PLYMORPHISM	NMD
rs61742596	NT5C1B	NOT FOUND	POSSIBLY PLYMORPHISM	NMD
rs61745064	DNAH14	DAMAGING	DISEASE CAUSING	NMD
rs61746769	RAET1E	DAMAGING	POSSIBLY PLYMORPHISM	NMD
rs61750839	SPTBN5	DAMAGING	DISEASE CAUSING	NMD
rs61751875	OR7G3	DAMAGING	POSSIBLY PLYMORPHISM	truncated protein (might cause NMD)
rs61753375	EME2	DAMAGING	DISEASE CAUSING	truncated protein (might cause NMD)
rs61816761	FLG	DAMAGING	DISEASE CAUSING	truncated protein (might cause NMD)
rs61887097	OR8I2	DAMAGING	POSSIBLY PLYMORPHISM	truncated protein (might cause NMD)
rs61942233	OAS3	DAMAGING	DISEASE CAUSING	NMD
rs61950356	SKA3	DAMAGING	DISEASE CAUSING	NMD
rs62154921	VWA3B	NOT FOUND	POSSIBLY PLYMORPHISM	NMD
rs62239058	SLC5A4	DAMAGING	DISEASE CAUSING	NMD
rs62623709	C1orf194	DAMAGING	DISEASE CAUSING	NMD
rs6671527	MOB3C	DAMAGING	POSSIBLY PLYMORPHISM	NMD
rs67047829	ERV3-1	DAMAGING	POSSIBLY PLYMORPHISM	truncated protein (might cause NMD)
rs6907580	GPRC6A	DAMAGING	POSSIBLY PLYMORPHISM	NMD
rs7120775	OR4X2	DAMAGING	POSSIBLY PLYMORPHISM	truncated protein (might cause NMD)
rs71358943	CYP2A7	DAMAGING	POSSIBLY PLYMORPHISM	truncated protein (might cause NMD)
rs71371463	KRTAP4-5	DAMAGING	DISEASE CAUSING	truncated protein (might cause NMD)
rs71377306	C17orf57	DAMAGING	POSSIBLY PLYMORPHISM	truncated protein (might cause NMD)
rs7218125	OR3A1	DAMAGING	DISEASE CAUSING	truncated protein (might cause NMD)
rs72856718	CCHCR1	NOT FOUND	POSSIBLY PLYMORPHISM	NMD
rs73366471	TYMS,C18orf56	DAMAGING	NOT FOUND	truncated protein (might cause NMD)
rs73474332	C7orf29	DAMAGING	DISEASE CAUSING	truncated protein (might cause NMD)
rs73489977	THEG	NOT FOUND	POSSIBLY PLYMORPHISM	NMD
rs73575237	POTEA	DAMAGING	NOT FOUND	truncated protein (might cause NMD)
rs74048215	C11orf21	DAMAGING	DISEASE CAUSING	NMD
rs74118444	RUSC1,RUSC1-AS1	DAMAGING	NOT FOUND	truncated protein (might cause NMD)
rs74437357	ATP13A5	DAMAGING	POSSIBLY PLYMORPHISM	NMD
rs7447815	SLC6A18	DAMAGING	POSSIBLY PLYMORPHISM	NMD
rs74697203	GP6	DAMAGING	DISEASE CAUSING	truncated protein (might cause NMD)
rs74830030	MATK	DAMAGING	POSSIBLY PLYMORPHISM	NMD
rs7485773	ACSM4	DAMAGING	POSSIBLY PLYMORPHISM	NMD
rs74969489	C17orf57	DAMAGING	DISEASE CAUSING	NMD
rs7499011	PKD1L2	DAMAGING	POSSIBLY PLYMORPHISM	NMD
rs75411676	PRAMEF2	DAMAGING	POSSIBLY PLYMORPHISM	NMD
rs75423534	OR4C11	DAMAGING	POSSIBLY PLYMORPHISM	truncated protein (might cause NMD)
rs75624587	OTOF	DAMAGING	POSSIBLY PLYMORPHISM	NMD
rs75656517	ABCA10	DAMAGING	DISEASE CAUSING	NMD
rs75898556	OR4D10	DAMAGING	POSSIBLY PLYMORPHISM	truncated protein (might cause NMD)
rs76101114	TPPP2	DAMAGING	POSSIBLY PLYMORPHISM	truncated protein (might cause NMD)
rs76160133	OR4P4	DAMAGING	POSSIBLY PLYMORPHISM	truncated protein (might cause NMD)
rs76294770	RAET1L	DAMAGING	DISEASE CAUSING	NMD
rs76330087	ATP6V1G3	DAMAGING	POSSIBLY PLYMORPHISM	NMD
rs76438938	KNG1	DAMAGING	POSSIBLY PLYMORPHISM	truncated protein (might cause NMD)
rs7692722	ZNF718	NOT FOUND	NOT FOUND	truncated protein (might cause NMD)
rs77002186	SLC22A24	DAMAGING	DISEASE CAUSING	NMD

rs7786505	PVRIG	DAMAGING	POSSIBLY PLYMORPHISM	NMD
rs7809642	NPSR1	NOT FOUND	PLYMORPHISM	truncated protein (might cause NMD)
rs78256866	OR4M2	DAMAGING	DISEASE CAUSING	truncated protein (might cause NMD)
rs78283108	SERPINA9	DAMAGING	DISEASE CAUSING	NMD
rs78408237	MRGPRX3	DAMAGING	PLYMORPHISM	truncated protein (might cause NMD)
rs78835222	OVOS2	DAMAGING	NOT FOUND	truncated protein (might cause NMD)
rs78990556	CPNE7	DAMAGING	DISEASE CAUSING	NMD
rs7904983	PKD2L1	DAMAGING	POSSIBLY PLYMORPHISM	NMD
rs79448530	C11orf40	DAMAGING	POSSIBLY PLYMORPHISM	NMD
rs79707842	SPERT	DAMAGING	POSSIBLY PLYMORPHISM	truncated protein (might cause NMD)
rs79802002	OR2W5	DAMAGING	NOT FOUND	truncated protein (might cause NMD)
rs79892855	ZNF80	DAMAGING	DISEASE CAUSING	truncated protein (might cause NMD)
rs79958632	PRSS45	DAMAGING	POSSIBLY PLYMORPHISM	NMD
rs80072371	SPERT	DAMAGING	POSSIBLY PLYMORPHISM	truncated protein (might cause NMD)
rs8072510	SLFN13	DAMAGING	POSSIBLY PLYMORPHISM	NMD
rs8108078	SIGLEC5	DAMAGING	POSSIBLY PLYMORPHISM	NMD
rs8192646	TAAR2	DAMAGING	POSSIBLY PLYMORPHISM	truncated protein (might cause NMD)
rs850763	SLC5A9	DAMAGING	POSSIBLY PLYMORPHISM	NMD
rs863362	OR10X1	DAMAGING	POSSIBLY PLYMORPHISM	truncated protein (might cause NMD)
rs877346	KRTAP13-2	DAMAGING	PLYMORPHISM	truncated protein (might cause NMD)
rs9427397	FCGR2A	DAMAGING	POSSIBLY PLYMORPHISM	NMD
rs9886752	LCN10	NOT FOUND	POSSIBLY PLYMORPHISM	NMD
rs9973206	USP29	DAMAGING	PLYMORPHISM	truncated protein (might cause NMD)
rs497116	CASP12	NOT FOUND	POSSIBLY PLYMORPHISM	NMD
rs1815739	ACTN3	DAMAGING	NOT FOUND	truncated protein (might cause NMD)

Table S2

Bioinformatics analysis of 246 PTCs with DAF >1% based on SIFT

246 PTC mutations were validated using a commonly used mutation program of pathogenicity, SIFT, which determine the degree of tolerance for each amino acid substitution on the basis of physiochemical properties.

Supplementary Methods

Multivariate analysis of variance

Multivariate analysis (Hierarchical clustering, Non-Hierarchical clustering, and Principal component analysis (PCA)) was carried out using the R 3.02 statistical software together with the Rcmdr package. Input matrices were 246 PTC mutations with DAF >1% (246x14 datasheets) from 14 ethnic groups of 1000G. Ward's algorithms were performed on the matrices of spectra from 14 ethnic groups to configure the setting for hierarchical clustering coefficient. The euclidean distance was computed to measure the pairwise profile similarity and obtain the dendrogram. Non-Hierarchical clustering approach was based on k-Means algorithm (clustering seeds = 10) and the results were averaged over 10 repetitions. PCA was also used in order to separate the data set into clusters. Loading profiles of the principal components (PC 1 and 2) were applied to visualize ethnic variability of PTC mutations. The graphs were generated with Microsoft Excel 2013.

Supplementary Note

Olfaction

Olfaction is characterized by a remarkable ability to detect and discriminate thousands of odorants (1). Accurate odor perception is based on input from hundreds of sensory neuronal types equipped with diverse olfactory receptors (ORs) (1,2). We detected 31 disappearing genes with DAF>1% that do not have intact ORFs. The number and the fraction of non-functional genes are larger than the ones previously reported (3). The human OR genes can clearly be divided into class I and class II genes, as was previously noted (2), but we cannot detect the any relationship between classification and disappearing genes. The ligands for ORs currently remain unknown due to the redundant nature of the combinatorial code for odorant identity (1). By assigning ligands to ORs, measuring the functional consequences of segregating polymorphisms *in vitro* and linking *in vitro* function to human behavior, these data will provide new insights into modern evolution of olfactory perception.

Solute Carrier

The solute-carrier (SLC) gene superfamily encodes membrane-bound transporters (4,5). The SLC superfamily comprises 55 gene families having over 365 putatively functional protein-coding genes (4,5). The gene products act as uniporters, symporters and antiporters, located in plasma cell membranes and organelle membranes (4,5). Transported substrates include amino acids, neurotransmitters, sugars, inorganic cations/anions, bile salts, thyroid hormone, and so on (4,5). The SLC family including five disappearing genes may share the substrates and signaling pathways in common (4,5), and these may have facilitated evolution of SLC family.

Keratin

In this analysis we showed that *KRT83* for type II hair keratin (6), six KRTAPs (7,8) and two FLGs (9,10) have reduced in the numbers very recently in the human evolution although an expanded KRTAP gene repertoire is found even among some primates. Keratins and their related genes are major components of the hair fiber, which is unique structure found on all mammals (6-10). High-frequency PTCs in Keratin, KRTAPs and FLGs could potentially explain the differing hair features of human lineages and the thinning of human body hair. And clothing, along with large climate

change or modern technology may be a one of the primary driving force of hair-related gene evolution and made the insulating properties of body hair obsolete.

Zinc finger

The human genome encodes over 700 zinc finger (ZNF) genes, which perform key roles in transcription process (11). The dramatic increase in the number of ZNF genes are observed from yeast to *Drosophila*, to *Xenopus* and to humans and gene duplication has facilitated the expansion of this family in eukaryotic lineages (12). Recent study of adaptive evolution among ZNF paralogs using d_N/d_S analysis shows that a major component of the selective pressure acting on ZNF genes has been positive selection to change their DNA-binding specificity (12). The reduction of number in ZNF genes we detected may be a sequence of ZNF evolution and thus driver for eukaryotic evolution.

Taste

Taste is a primitive sense that helps detect and distinguish key dietary components along with olfaction (13). The sensation of taste is classified into five prototypical categories: sweet, bitter, sour, umami, and salty (13). In addition recent studies raise the possibility of an additional sixth and seventh taste modality devoted to the perception of fats (14) and calcium (15). We have recently showed that modern humans may have been losing their taste receptors and molecular evolutionary rates of sour and bitter receptors were considerably higher in humans than those of sweet, umami, and salty receptors in contrast to other carnivorous mammals (16). Much is still unknown about relationships among their genetic variants, dietary choices, and the signal transmission pathways. Further biochemical studies are required to clarify the relationship between modern human’s taste and genetic variants.

Spermatogenesis

Spermatogenesis is a long and complex process that, despite the shared overall goal of forming the male gamete, displays striking amounts of interspecific diversity (17). The product of many genes play important roles in various aspects of spermatogenesis (18). Spermatogenesis-associated (SPATA) family we detected is

necessary for the formation of the sperm acrosome (19,20). DNAH family is involved in sperm motility and part of the sperm flagella assembly (21). A greater increase in their rate of evolution is observed relative to genes expressed in other tissues and stronger sexual selection pressure acting on these genes (17). The reduced number of genes associated with spermatogenesis have likely followed this tendency even during human evolution.

Drug metabolism

Recent pharmacogenomic researches have revealed extensive variations in drug-metabolizing capacity among individuals (22,23). The main indexes for this variation in drug metabolism are variability of UDP-glucuronosyltransferase and Cytochrome P450 genes, which have been proven to be highly polymorphic (22,23,24). Such polymorphisms have been shown to be of importance for causing adverse effects or lack of therapeutic efficacy (22,23,24). Our results raise the possibility that modern humans might have been losing their drug-metabolizing ability. Modern life's evolutionary pressures, such as improvement of diets, irritants and toxicant, may be hastening the human CYP evolution.

RNA virus

The endogenous viral elements (EVE) are DNA sequences derived from retrovirus or non-retrovirus (25,26). More than 10% of the human genome is made up of these elements (25,26). Despite recent advances in studies of EVEs, their function is still unknown. Here we detected four EVEs: *EBLN2* is non-retroviral origin, and *ERVMER34-1*, *ERV3-1* and *HHLA3* are retroviral ones.

Ligand for NGK2D

Six ULBP/RAET (UL16-binding protein, also known as retinoic acid early transcript) genes encode ligands of the activating immunoreceptor NKG2D and are associated with NK-cell activation (27,28). Out of six genes, *ULBP6/RAET1L*, *ULBP4/RAET1E* and *ULBP3/RAET1N* are frequently mutated. It is expected that there is a functional redundancy among a set of NKG2D ligands.

Interferon

Interferons (IFNs) are helicoidal cytokines released by host cells in response to the presence of pathogens or tumor cells (29). Based on the type of receptor through which they signal, human IFNs have been classified into three major types (from Type I to III) (29). We detected two type I INFs (*IFNA10* and *IFNE*) as having DAF >1% of PTC mutation. Type I IFNs include a total of 17 subtypes (13 subtypes of IFN-α and IFNs β/ε/κ/ω), all of which bind to a receptor composed of two chains, *IFNAR1* and *IFNAR2* (30). Type I INFs are characterized by considerable functional redundancy and pleiotropism, and thus modern humans may have begun disposing some kinds of INFs.

Immune defense receptors

CLEC7A/Dectin-1 is shown to recognize several fungal genera, including *Candida*, *Aspergillus*, *Pneumocystis*, and others, and its deficiency (OMIM *606264; rs16910526, p.Tyr238*) is associated with mucocutaneous fungal infections (31,32). A common dominant *TLR5* nonsense mutation (OMIM *603031; rs5744168, p.Arg392*) has been shown to susceptibility to *Legionnaire* and *Aspergillosis* disease and Melioidosis (33,34). *LGSLA8/Galectin-8*, a cytosolic lectin, is a danger receptor that restricts *Salmonella* proliferation (35). The frequency nonsense codons in immune

defense receptors raises the question of whether there is an evolutionary advantage to having these genetic variants. One rational explanation for this is that these mutations provides protection against autoimmune disease as one of evolutionary pressures. This hypothesis is supported by the previous report that *TLR5* PTC mutation leads to resistance to systemic lupus erythematosus (SLE) (36). To make a negative interpretation we guess that modern humans may have physically weaker and more susceptible to pathogens.

Other gene family

The POTE family genes encode a highly homologous group of primate-specific proteins that contain ankyrin repeats and coiled coil domains and is induced in many cancers (e.g. prostate cancer) (37,38). The MAGE (melanoma-associated antigen) gene family was originally identified as “a melanoma antigen” and share a homologous MAGE conserved domain located close to the C-terminal (39,40). The acyl-CoA synthetase medium-chain (ACSM) family encode for enzymes catalyzing the activation of medium-chain length fatty acids (41,42) and is associated with plasma high-density lipoprotein cholesterol levels (42). Although the function of these family is largely

unknown, we conclude that PTC mutations have a strong tendency to be introduced to the members of the same gene family even during human evolution.

References in Supplementary note

1. DeMaria, S. & Ngai, J. The cell biology of smell. The Journal of cell biology 191, 443-452, doi:10.1083/jcb.201008163 (2010)

2. Glusman, G., Yanai, I., Rubin, I. & Lancet, D. The complete human olfactory subgenome. Genome Res 11, 685-702, doi:10.1101/gr.171001 (2001)

3. Hughes GM, Teeling EC, Higgins DG (2014) Loss of Olfactory Receptor Function in Hominin Evolution. PLoS ONE 9(1): e84714.

4. He, L., Vasiliou, K. & Nebert, D. W. Analysis and update of the human solute carrier (SLC) gene superfamily. Hum Genomics 3, 195-206 (2009)

5. Hediger, M. A. et al. The ABCs of solute carriers: physiological, pathological and therapeutic implications of human membrane transport proteins. Pflugers Archiv 447, 465-468, doi:10.1007/s00424-003-1192-y (2004)

6. Schweizer, J. et al. New consensus nomenclature for mammalian keratins. The Journal of cell biology 174, 169-174, doi:10.1083/jcb.200603161 (2006)

7. Khan, I. et al. Mammalian keratin associated proteins (KRTAPs) subgenomes: disentangling hair diversity and adaptation to terrestrial and aquatic environments. BMC genomics 15, 779, doi:10.1186/1471-2164-15-779 (2014)

8. Wu, D. D., Irwin, D. M. & Zhang, Y. P. Molecular evolution of the keratin associated protein gene family in mammals, role in the evolution of mammalian hair. BMC evolutionary biology 8, 241, doi:10.1186/1471-2148-8-241 (2008)

9. Brown, S. J. & McLean, W. H. One remarkable molecule: filaggrin. The Journal of investigative dermatology 132, 751-762, doi:10.1038/jid.2011.393 (2012)

10. Makino, T., Mizawa, M., Yamakoshi, T., Takaishi, M. & Shimizu, T. Expression of filaggrin-2 protein in the epidermis of human skin diseases: a comparative analysis with filaggrin. Biochemical and biophysical research communications 449, 100-106, doi:10.1016/j.bbrc.2014.04.165 (2014)

11. Nowick, K. et al. Gain, loss and divergence in primate zinc-finger genes: a rich resource for evolution of gene regulatory differences between species. PloS one 6, e21553, doi:10.1371/journal.pone.0021553 (2011)

12. Emerson, R. O. & Thomas, J. H. Adaptive evolution in zinc finger transcription factors. PLoS genetics 5, e1000325, doi:10.1371/journal.pgen.1000325 (2009)

13. Yarmolinsky, D. A., Zuker, C. S. & Ryba, N. J. Common sense about taste: from mammals to insects. Cell 139, 234-244, doi:10.1016/j.cell.2009.10.001 (2009)

14. Laugerette, F. et al. CD36 involvement in orosensory detection of dietary lipids, spontaneous fat preference, and digestive secretions. The Journal of clinical investigation 115, 3177-3184, doi:10.1172/JCI25299 (2005)

15. Tordoff, M. G., Alarcon, L. K., Valmeki, S. & Jiang, P. T1R3: a human calcium taste receptor. Scientific reports 2, 496, doi:10.1038/srep00496 (2012)

16. Fujikura, K. Multiple loss-of-function variants of taste receptors in modern human populations. Scientific reports 5, 12349 (2015).

17. Ramm, S. A., Scharer, L., Ehmcke, J. & Wistuba, J. Sperm competition and the evolution of spermatogenesis. Molecular human reproduction, doi:10.1093/molehr/gau070 (2014)

18. Johnston, D. S. et al. Stage-specific gene expression is a fundamental characteristic of rat spermatogenic cells and Sertoli cells. Proceedings of the

National Academy of Sciences of the United States of America 105, 8315-8320, doi:10.1073/pnas.0709854105 (2008)

19. Nie, D. & Xiang, Y. Molecular cloning and characterization of a novel human testis-specific gene by use of digital differential display. Journal of genetics 85, 57-62 (2006)

20. Chen, H., Yi, M., Sheng, Y., Cheng, H. & Zhou, R. A novel testis-enriched gene Spata33 is expressed during spermatogenesis. PloS one 8, e67882, doi:10.1371/journal.pone.0067882 (2013)

21. Pfister, K.K., Shah, P.R., Hummerich, H., Russ, A., Cotton, J., et al. Genetic Analysis of the Cytoplasmic Dynein Subunit Families. PLoS Genetics 2, e1 (2006) doi:10.1371/journal.pgen.0020001

22. Nebert, D. W. & Russell, D. W. Clinical importance of the cytochromes P450. Lancet 360, 1155-1162, doi:10.1016/S0140-6736(02)11203-7 (2002)

23. Sim, S. C. & Ingelman-Sundberg, M. The Human Cytochrome P450 (CYP) Allele Nomenclature website: a peer-reviewed database of CYP variants and their associated effects. Human genomics 4, 278-281 (2010)

24. Guillemette, C. Pharmacogenomics of human UDP-glucuronosyltransferase enzymes. The pharmacogenomics journal 3, 136-158, doi:10.1038/sj.tpj.6500171 (2003)

25. Katzourakis, A. & Gifford, R. J. Endogenous viral elements in animal genomes. PLoS genetics 6, e1001191, doi:10.1371/journal.pgen.1001191 (2010)

26. Feschotte C, Gilbert C. Endogenous viruses: insights into viral evolution and impact on host biology. Nat Rev Genet. 2012 Mar 16;13(4):283-96. doi: 10.1038/nrg3199.

27. González S, López-Soto A, Suarez-Alvarez B, López-Vázquez A, López-Larrea C. NKG2D ligands: key targets of the immune response. Trends Immunol. 2008 Aug;29(8):397-403. doi: 10.1016/j.it.2008.04.007. Epub 2008 Jul 3.

28. Champsaur, M. & Lanier, L. L. Effect of NKG2D ligand expression on host immune responses. Immunological reviews 235, 267-285, doi:10.1111/j.0105-2896.2010.00893.x (2010)

29. Manry, J. et al. Evolutionary genetic dissection of human interferons. The Journal of experimental medicine 208, 2747-2759, doi:10.1084/jem.20111680 (2011)

30. Uze, G., Schreiber, G., Piehler, J. & Pellegrini, S. The receptor of the type I interferon family. Current topics in microbiology and immunology 316, 71-95 (2007)

31. Ferwerda, B. et al. Human dectin-1 deficiency and mucocutaneous fungal infections. The New England journal of medicine 361, 1760-1767, doi:10.1056/NEJMoa0901053 (2009)

32. Saijo, S. et al. Dectin-1 is required for host defense against Pneumocystis carinii but not against Candida albicans. Nature immunology 8, 39-46, doi:10.1038/ni1425 (2007)

33. Hawn, T. R. et al. A common dominant TLR5 stop codon polymorphism abolishes flagellin signaling and is associated with susceptibility to legionnaires' disease. The Journal of experimental medicine 198, 1563-1572, doi:10.1084/jem.20031220 (2003)

34. West, T. E. et al. Impaired TLR5 functionality is associated with survival in melioidosis. Journal of immunology 190, 3373-3379, doi:10.4049/jimmunol.1202974 (2013)

35. Thurston, T. L., Wandel, M. P., von Muhlinen, N., Foeglein, A. & Randow, F. Galectin 8 targets damaged vesicles for autophagy to defend cells against bacterial invasion. Nature 482, 414-418, doi:10.1038/nature10744 (2012)

36. Hawn, T. R. et al. A stop codon polymorphism of Toll-like receptor 5 is associated with resistance to systemic lupus erythematosus. Proceedings of the National Academy of Sciences of the United States of America 102, 10593-10597, doi:10.1073/pnas.0501165102 (2005)

37. Bera, T. K. et al. POTE paralogs are induced and differentially expressed in many cancers. Cancer research 66, 52-56, doi:10.1158/0008-5472.CAN-05-3014 (2006)

38. Ise, T. et al. Expression of POTE protein in human testis detected by novel monoclonal antibodies. Biochemical and biophysical research communications 365, 603-608, doi:10.1016/j.bbrc.2007.10.195 (2008)

39. Katsura, Y. & Satta, Y. Evolutionary history of the cancer immunity antigen MAGE gene family. PloS one 6, e20365, doi:10.1371/journal.pone.0020365 (2011)

40. Zhao, Q., Caballero, O. L., Simpson, A. J. & Strausberg, R. L. Differential evolution of MAGE genes based on expression pattern and selection pressure. PloS one 7, e48240, doi:10.1371/journal.pone.0048240 (2012)

41. Watkins, P. A., Maiguel, D., Jia, Z. & Pevsner, J. Evidence for 26 distinct acyl-coenzyme A synthetase genes in the human genome. Journal of lipid research 48, 2736-2750, doi:10.1194/jlr.M700378-JLR200 (2007)

42. Haketa, A. et al. Two medium-chain acyl-coenzyme A synthetase genes, SAH and MACS1, are associated with plasma high-density lipoprotein cholesterol levels, but they are not associated with essential hypertension. Journal of hypertension 22, 1903-1907 (2004)
